# Supplementary material for: X-linked myopathy with excessive autophagy: characterization and therapy testing in a zebrafish model
Source: EMBO Mol Med. 2025 Feb 24;17(4):823–40. doi: 10.1038/s44321-025-00204-8 (PMC11982336; doi:10.1038/s44321-025-00204-8)
Supplement: Supplementary file 1 — Appendix [file 44321_2025_204_MOESM1_ESM.pdf]

Appendix

Appendix Table S1 (p. 2)

Appendix Table S1: n and p values

Figure	subsection	n value per group	p value	Statistical Test performed
1	D	30 per group with 3 biological replicates	Siblings vs vma21Δ1/Δ1 = 0.0033<br>Siblings vs vma21Δ14ins21/ Δ14ins21 = 0.0004	unpaired two-tailed *t*-test
2	E	WT= 9,<br>HETS= 9,<br>Mutants= 13	WT vs Hets= 0.9846<br>WT vs mutants= 0.0415<br>Hets vs mutants= 0.0616	one-way ANOVA with Tukey’s multiple comparisons test
2	F	WT= 10<br>Mutants= 13	<0.0001	Fisher’s exact test
2	G	Siblings = 194<br>vma21Δ1/Δ1 n=74,<br>vma21Δ14ins21/Δ14ins21 n=60,<br>vma21Δ1/Δ14ins21 n=60.	<0.0001	Mantel Cox test
2	H	n=24/group with 3 replicates	Total time spent moving= <0.0001<br>Total distance travelled=<0.0001<br>Average total velocity= <0.0001	unpaired two-tailed t-test
2	I	n=24/group with 3 replicates	Total time spent moving= <0.0001<br>Total distance travelled=<0.0001<br>Average total velocity= <0.0001	unpaired two-tailed t-test
2	J	n=24/group with 3 replicates	Total time spent moving= <0.0001<br>Total distance travelled=<0.0001<br>Average total velocity= <0.0001	unpaired two-tailed t-test
2	K	n=24/group with 3 replicates	Total time spent moving= <0.0001<br>Total distance travelled=<0.0001<br>Average total velocity= 0.8269	unpaired two-tailed t-test
2	L	n=24/group with 3 replicates	Total time spent moving= <0.0001<br>Total distance travelled=<0.0001<br>Average total velocity= <0.0001	unpaired two-tailed t-test
2	M	n=24/group with 3 replicates	Total time spent moving= <0.0001<br>Total distance travelled=<0.0001<br>Average total velocity= <0.0001	unpaired two-tailed t-test
3	C	n=3 /group	0.0441	unpaired two-tailed t-test
3	G	WT= 16<br>Mutants= 8	0.0009	unpaired two-tailed t-test
3	H	WT= 11<br>Mutants= 15	0.0018	npaired two-tailed t-test
4	D	n = 10/group with 3 biological replicates	LC3 I (Siblings vs. vma21Δ14ins21/Δ14ins21)=<0.0001<br>LC3 I (Siblings vs. vma21Δ1/Δ14ins21)= 0.0009<br>LC3 II (Siblings vs. vma21Δ1/Δ1) = 0.0001<br>LC3 II (Siblings vs. vma21Δ14ins21/Δ14ins21)=0.0219<br>LC3 II (Siblings vs. vma21Δ1/Δ14ins21)=0.0294<br>LC3 II/ LC3 I ratio (Siblings vs. vma21Δ1/Δ1)= 0.0002<br>LC3 II/LC3 I ratio (Siblings vs.<br>vma21Δ14ins21/Δ14ins21)= 0.0002<br>LC3 II/LC3 I ratio (Siblings vs.	One-way ANOVA (with Dunnett's multiple comparisons test)
4	I	Siblings = 17<br>Mutants = 15	0.0002	unpaired two-tailed t-test
5	A-B	WT=6<br>Mutants = 6	n/a	n/a
5	E	cas9 only controls= 30<br>Mutants = 27	<0.0001	unpaired two-tailed t-test
5	H	WT= 29<br>Mutants= 26	<0.0001	Fisher’s exact test
6	A	n=100/group with 3 independent trials	n/a	n/a
6	B	n=100/group with 3 independent trials	n/a	n/a
6	C	n=100/group with 3 independent trials	n/a	n/a
6	D	Mutants (DMSO)= 10<br>Mutants (Edavarone) = 15<br>Mutants (LY294002)= 22	DMSO vs Edavarone = >0.9999<br>DMSO vs LY294002 = 0.6125	Fisher’s exact test
EV1	B	30 per group with 5 biological replicates	EtOH dose (0 vs 0.05)=0.0615<br>EtOH dose (0 vs 0.5) = <0.0001<br>EtOH dose (0 vs 1) = 0.1507	One-way ANOVA (with Dunnett's multiple comparisons test)
EV1	C	30 per group with 5 biological replicates	EtOH dose (0 vs 0.05)=0.9261<br>EtOH dose (0 vs 0.5) = 0.0918<br>EtOH dose (0 vs 1) = 0.3184	One-way ANOVA (with Dunnett's multiple comparisons test)
EV1	D	30 per group with 5 biological replicates	EtOH dose (0 vs 0.05)=0.0020<br>EtOH dose (0 vs 0.5) = <0.0001<br>EtOH dose (0 vs 1) = <0.0001	One-way ANOVA (with Dunnett's multiple comparisons test)

EV2	B	30 per group with 3 biological replicates	Untreated vs Vehicle = <0.0001<br>Untreated vs Edaravone= 0.1102<br>Untreated vs LY294002= <0.0001<br>Untreated vs GNE-7915 = <0.0001<br>Untreated vs CZC-25146 = <0.0001<br>Untreated vs Lucanthone = >0.9999<br>Untreated vs MRT68921 HCl= <0.0001<br>Untreated vs DC661 = <0.0001<br>Untreated vs GSK2578215A = <0.0001<br>Untreated vs ROC-325= <0.0001	One-way ANOVA (with Dunnett's multiple comparisons test)
EV2	D	30 per group with 3 biological replicates	Untreated vs Vehicle = 0.1423<br>Untreated vs Edaravone= <0.0001<br>Untreated vs LY294002= <0.0001<br>Untreated vs GNE-7915 = >0.9999<br>Untreated vs CZC-25146 = 0.9997<br>Untreated vs Lucanthone = 0.9997<br>Untreated vs MRT68921 HCl= 0.1153<br>Untreated vs DC661 = 0.8664<br>Untreated vs GSK2578215A = <0.0001<br>Untreated vs ROC-325= <0.0001	One-way ANOVA (with Dunnett's multiple comparisons test)
EV2	F	30 per group with 3 biological replicates	Untreated vs Vehicle = >0.9999<br>Untreated vs Edaravone= <0.0001<br>Untreated vs LY294002= <0.0001<br>Untreated vs GNE-7915 = <0.0001<br>Untreated vs CZC-25146 = 0.0496<br>Untreated vs Lucanthone = 0.0048<br>Untreated vs MRT68921 HCl= 0.0012<br>Untreated vs DC661 = 0.4284<br>Untreated vs GSK2578215A = 0.9997<br>Untreated vs ROC-325= >0.9999	One-way ANOVA (with Dunnett's multiple comparisons test)
