# Supplementary figures and images for: X-linked myopathy with excessive autophagy: characterization and therapy testing in a zebrafish model
Source: EMBO Mol Med. 2025 Feb 24;17(4):823–40. doi: 10.1038/s44321-025-00204-8 (PMC11982336; doi:10.1038/s44321-025-00204-8)

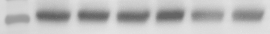

Supplement: Supplementary file 3 — Source data Fig. 1 [file 44321_2025_204_MOESM3_ESM.zip › Figure 1/1C- b-actin blot.tif]

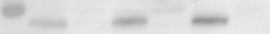

Supplement: Supplementary file 3 — Source data Fig. 1 [file 44321_2025_204_MOESM3_ESM.zip › Figure 1/1C- vma21 blot.tif]

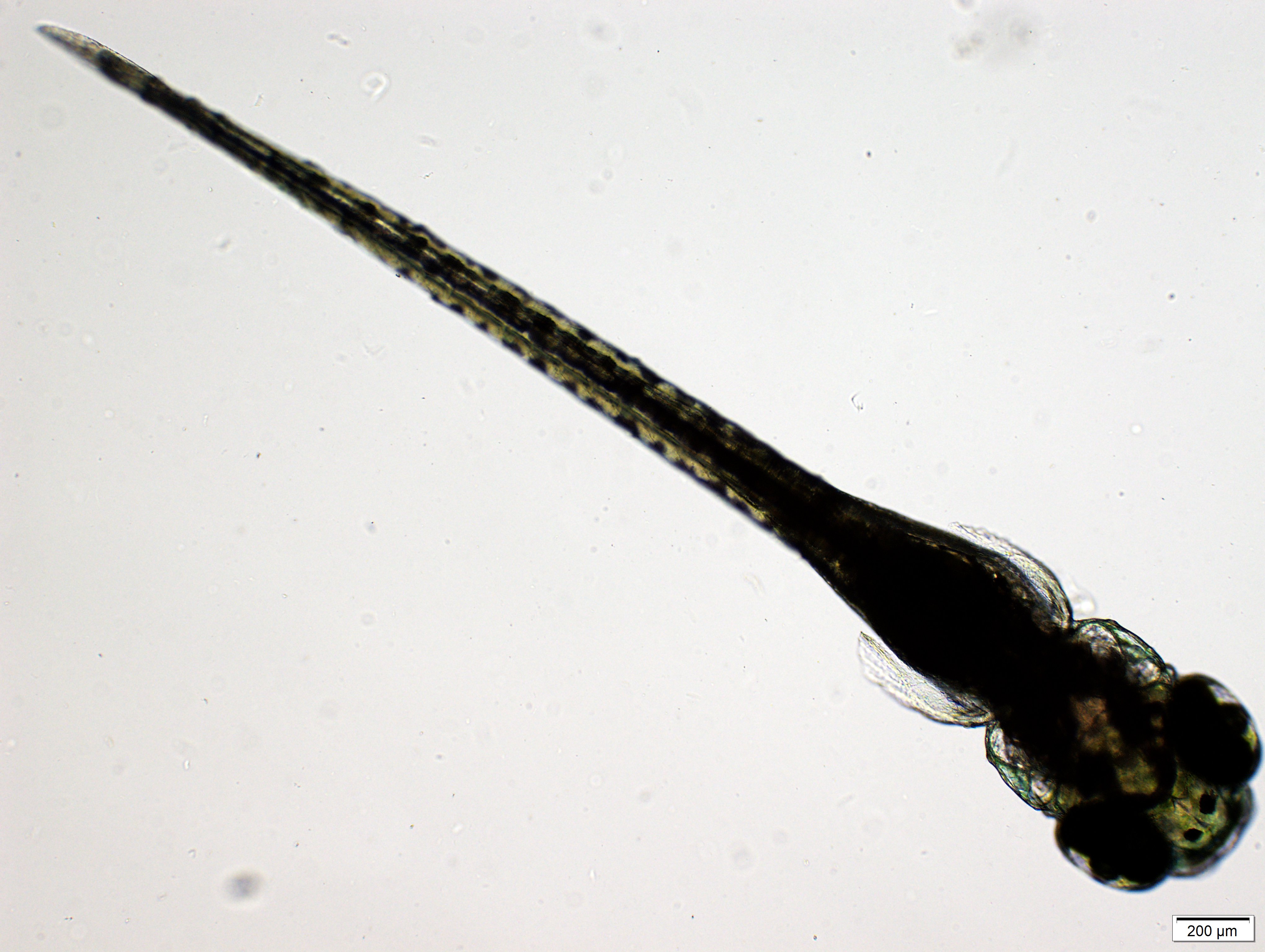

Supplement: Supplementary file 4 — Source data Fig. 2 [file 44321_2025_204_MOESM4_ESM.zip › Figure 2/2A- WT/2A- dorsal view.jpg]

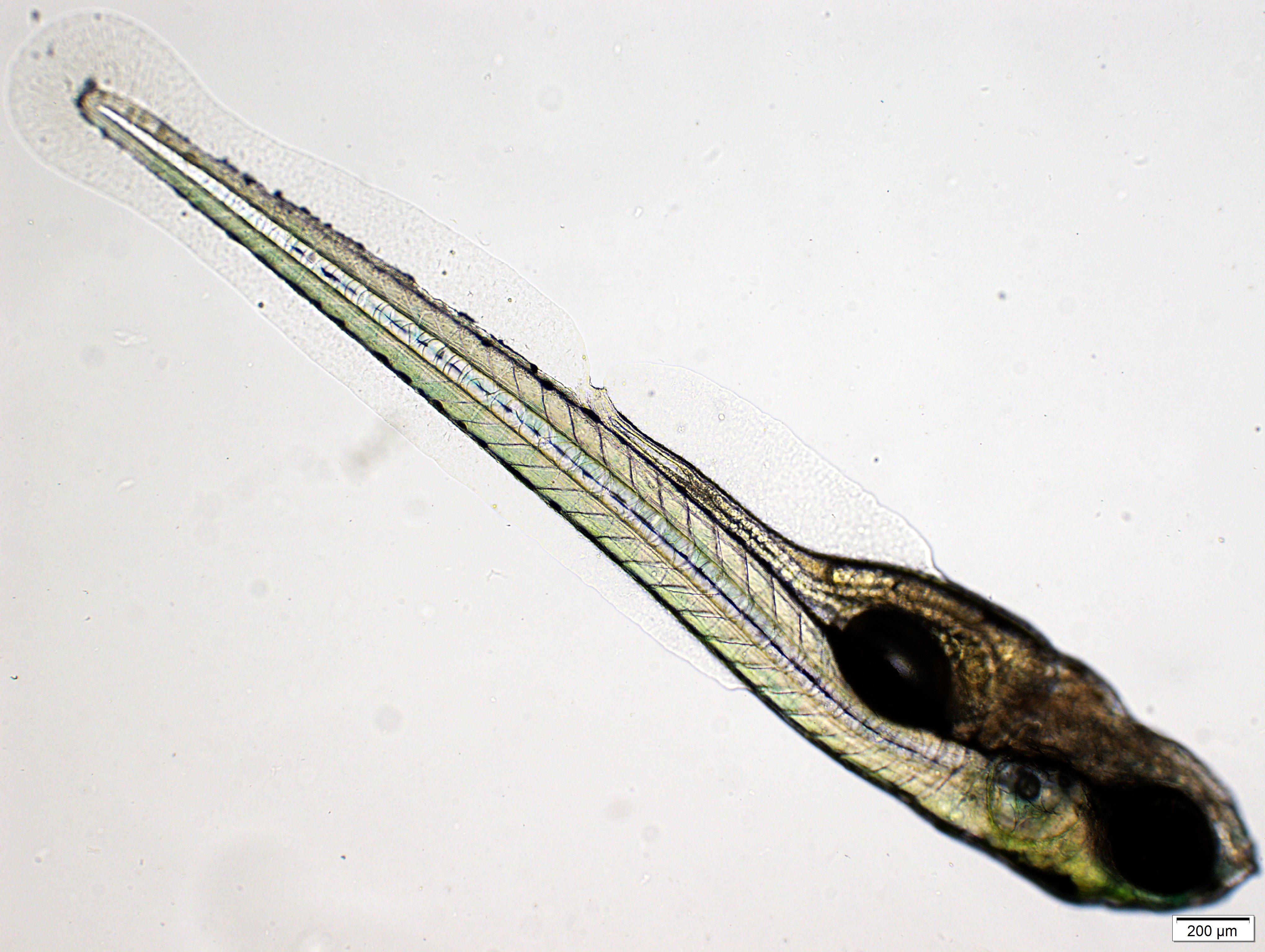

Supplement: Supplementary file 4 — Source data Fig. 2 [file 44321_2025_204_MOESM4_ESM.zip › Figure 2/2A- WT/2A- Lateral view.jpg]

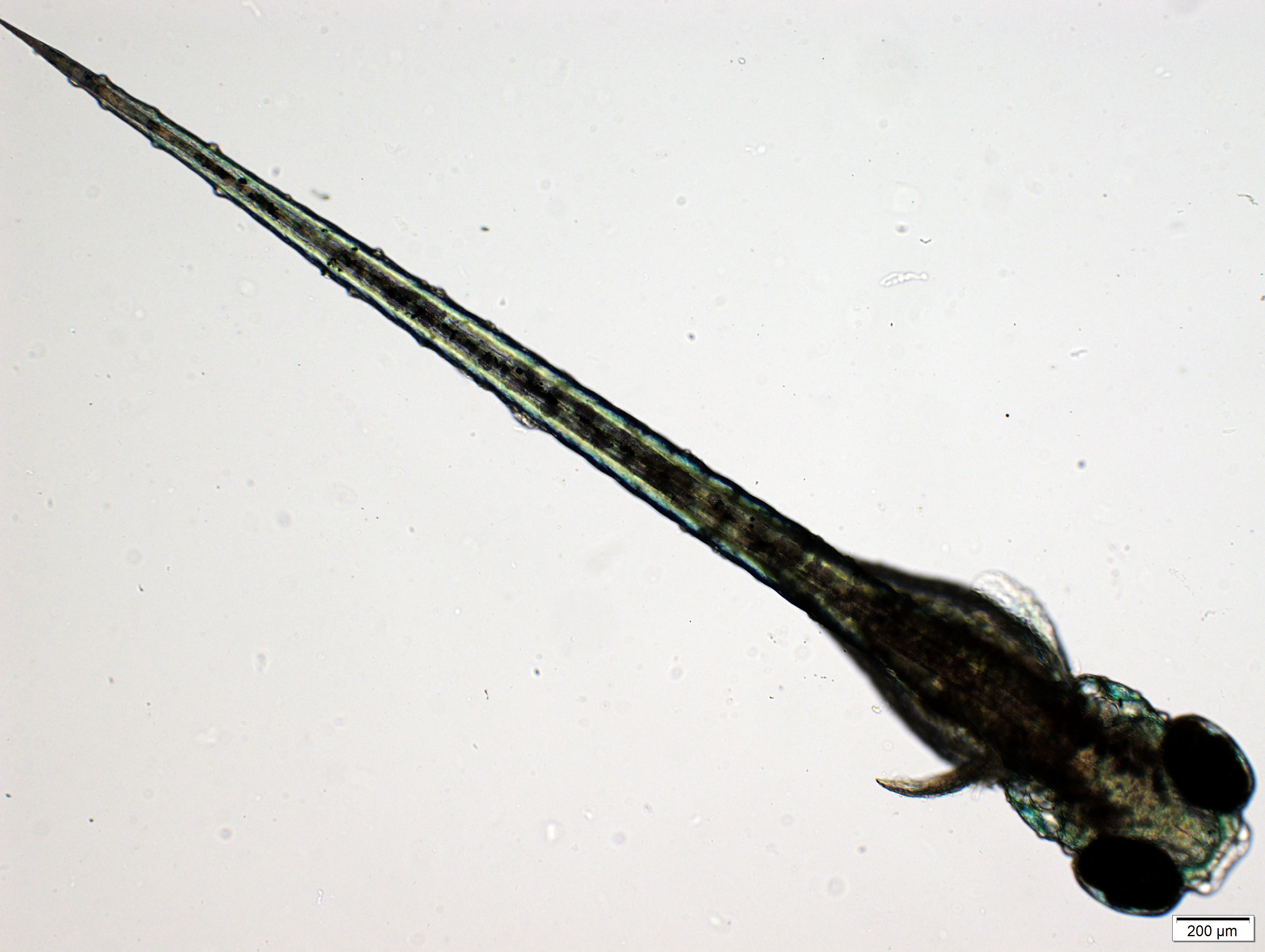

Supplement: Supplementary file 4 — Source data Fig. 2 [file 44321_2025_204_MOESM4_ESM.zip › Figure 2/2B/2B- dorsal view.jpg]

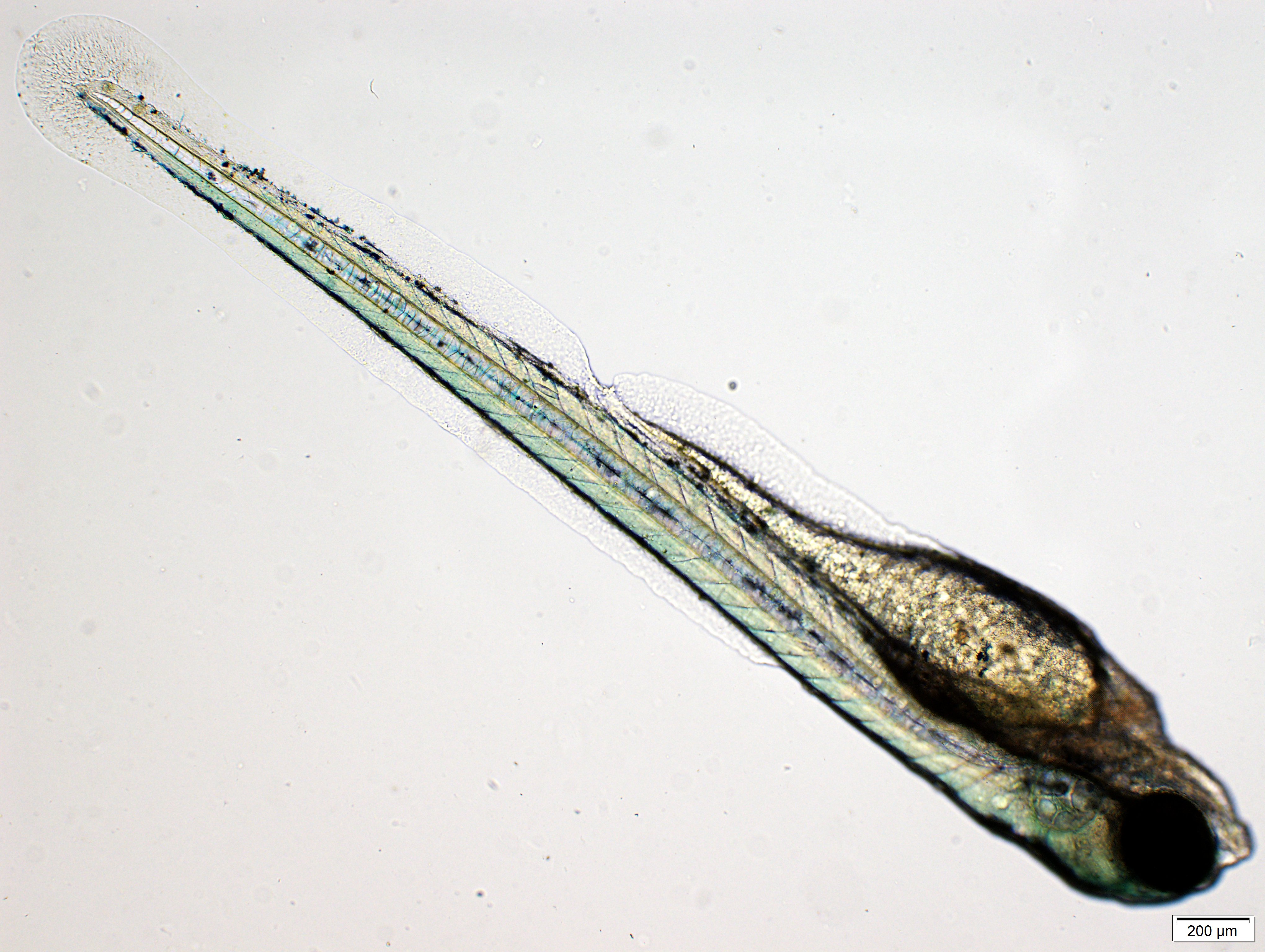

Supplement: Supplementary file 4 — Source data Fig. 2 [file 44321_2025_204_MOESM4_ESM.zip › Figure 2/2B/2B-Lateral view.jpg]

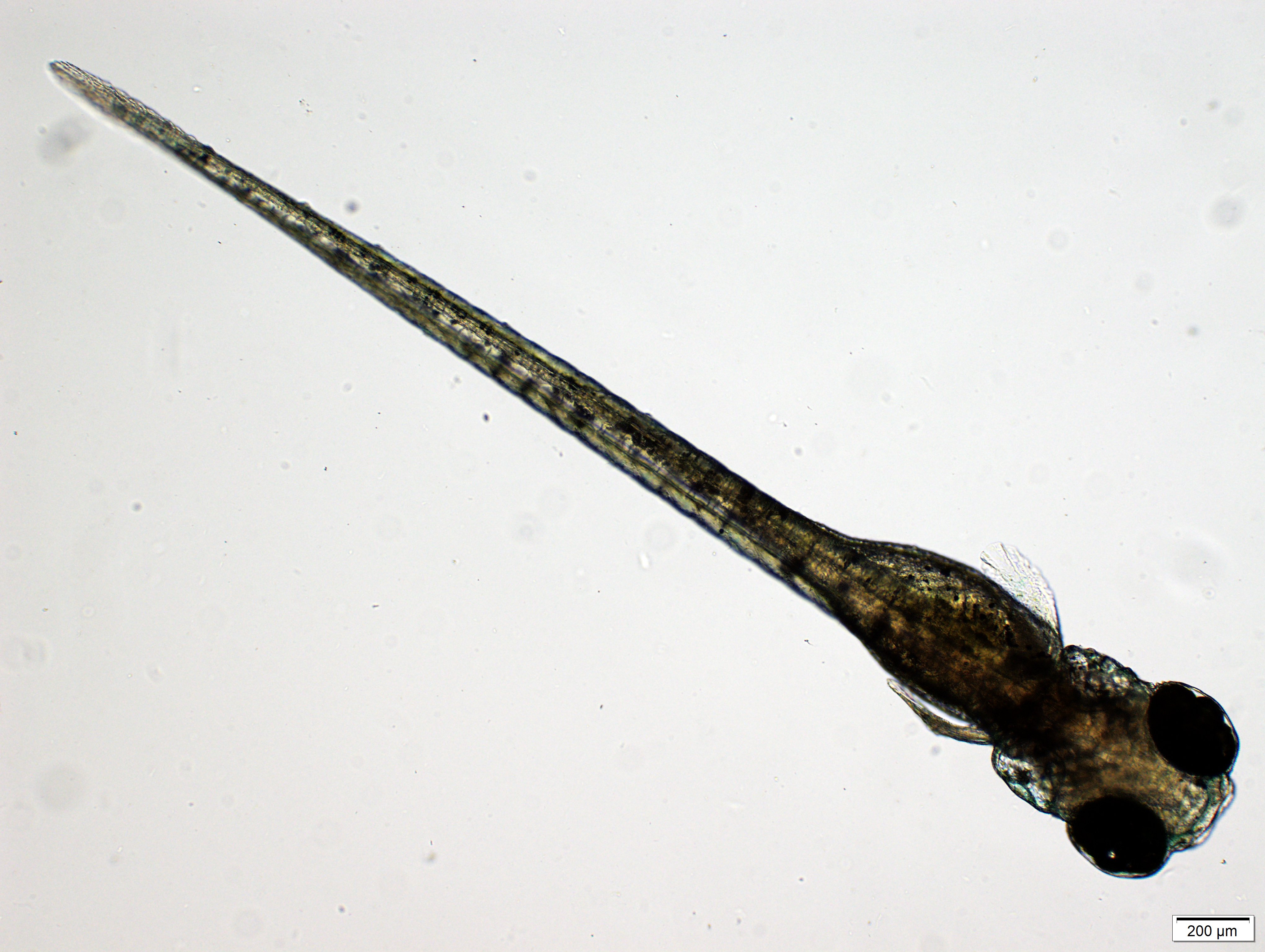

Supplement: Supplementary file 4 — Source data Fig. 2 [file 44321_2025_204_MOESM4_ESM.zip › Figure 2/2C/2C-Dorsal view.jpg]

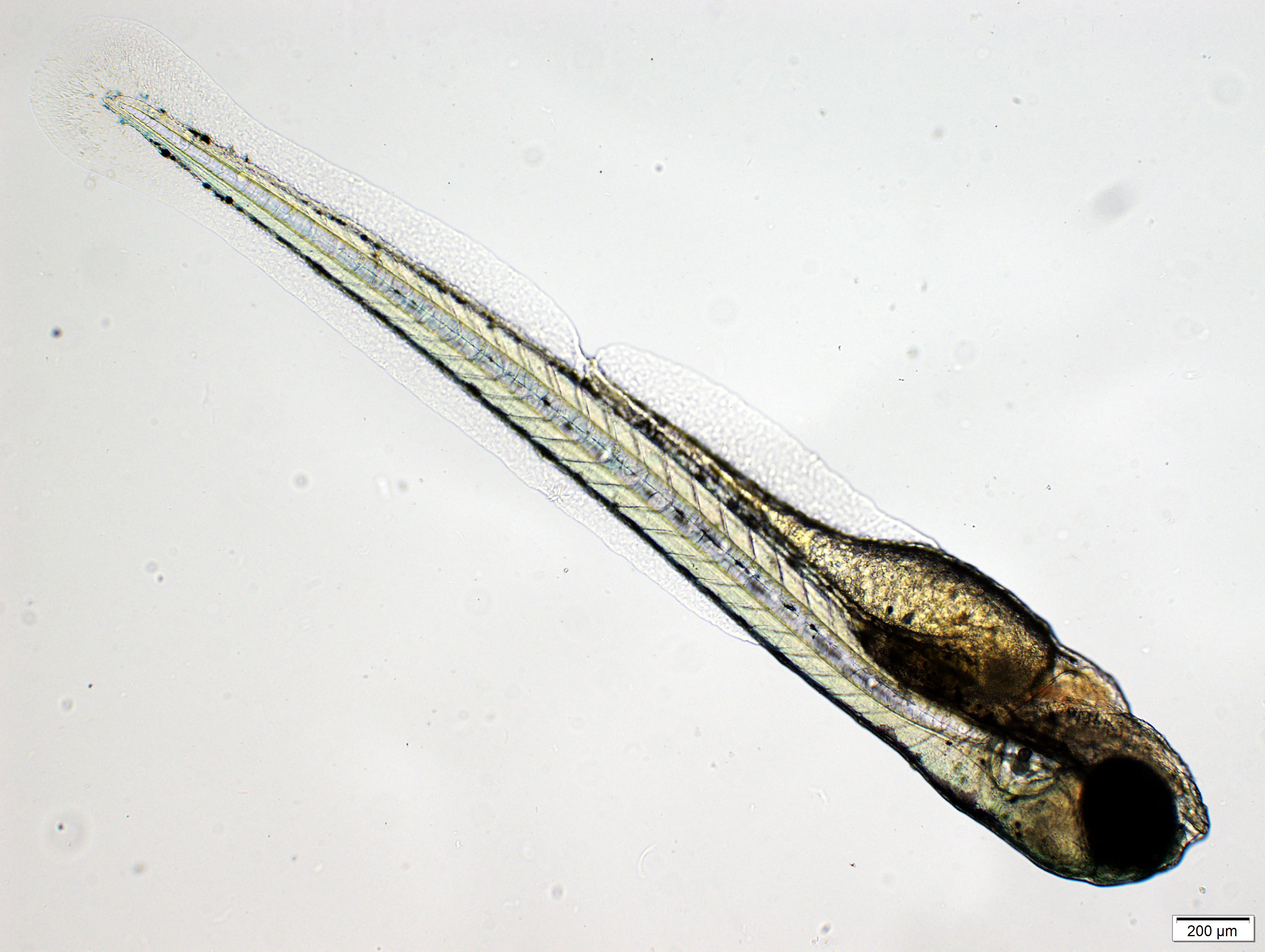

Supplement: Supplementary file 4 — Source data Fig. 2 [file 44321_2025_204_MOESM4_ESM.zip › Figure 2/2C/2C-Lateral view.jpg]

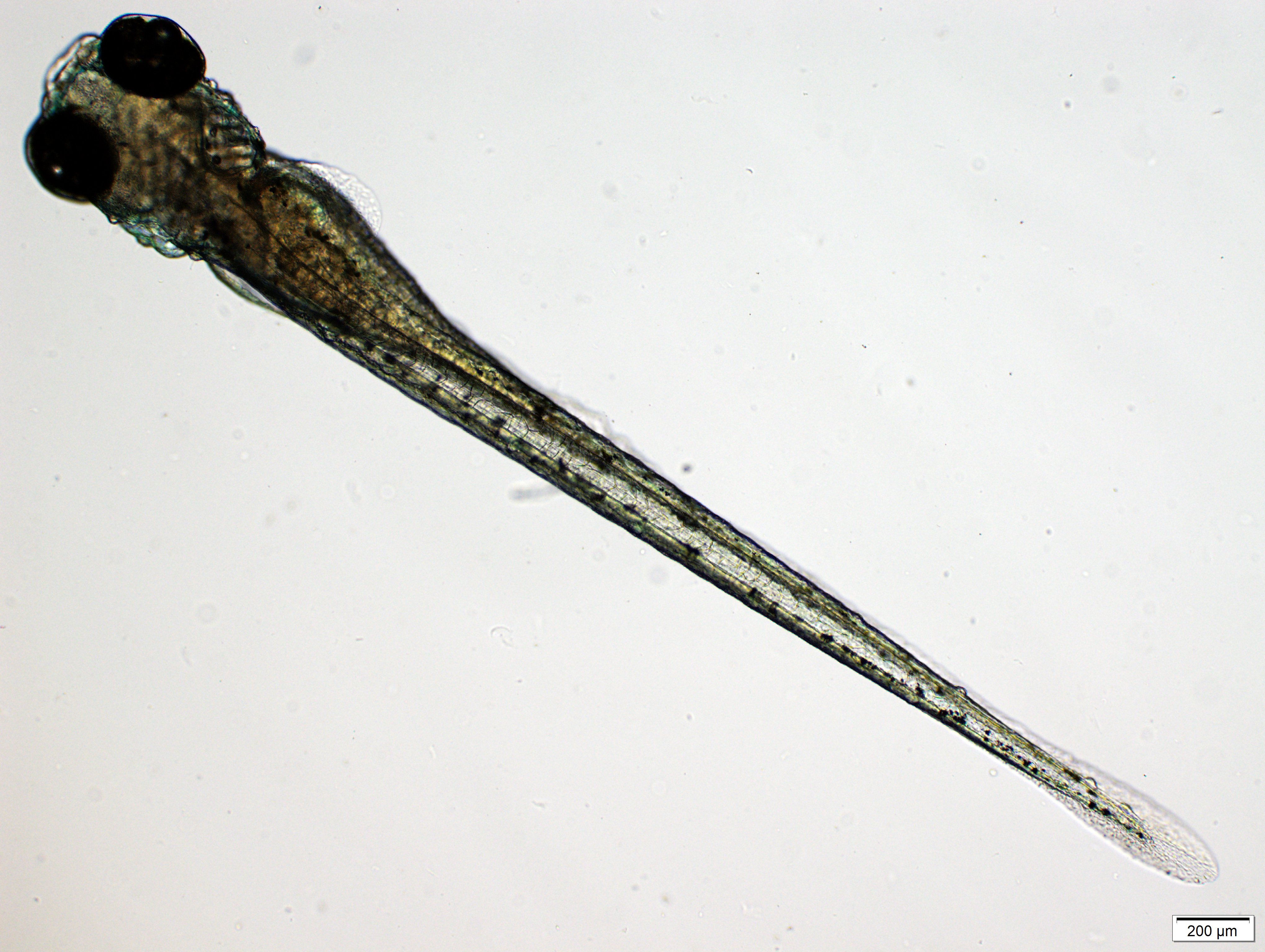

Supplement: Supplementary file 4 — Source data Fig. 2 [file 44321_2025_204_MOESM4_ESM.zip › Figure 2/2D/2D-Dorsal view.jpg]

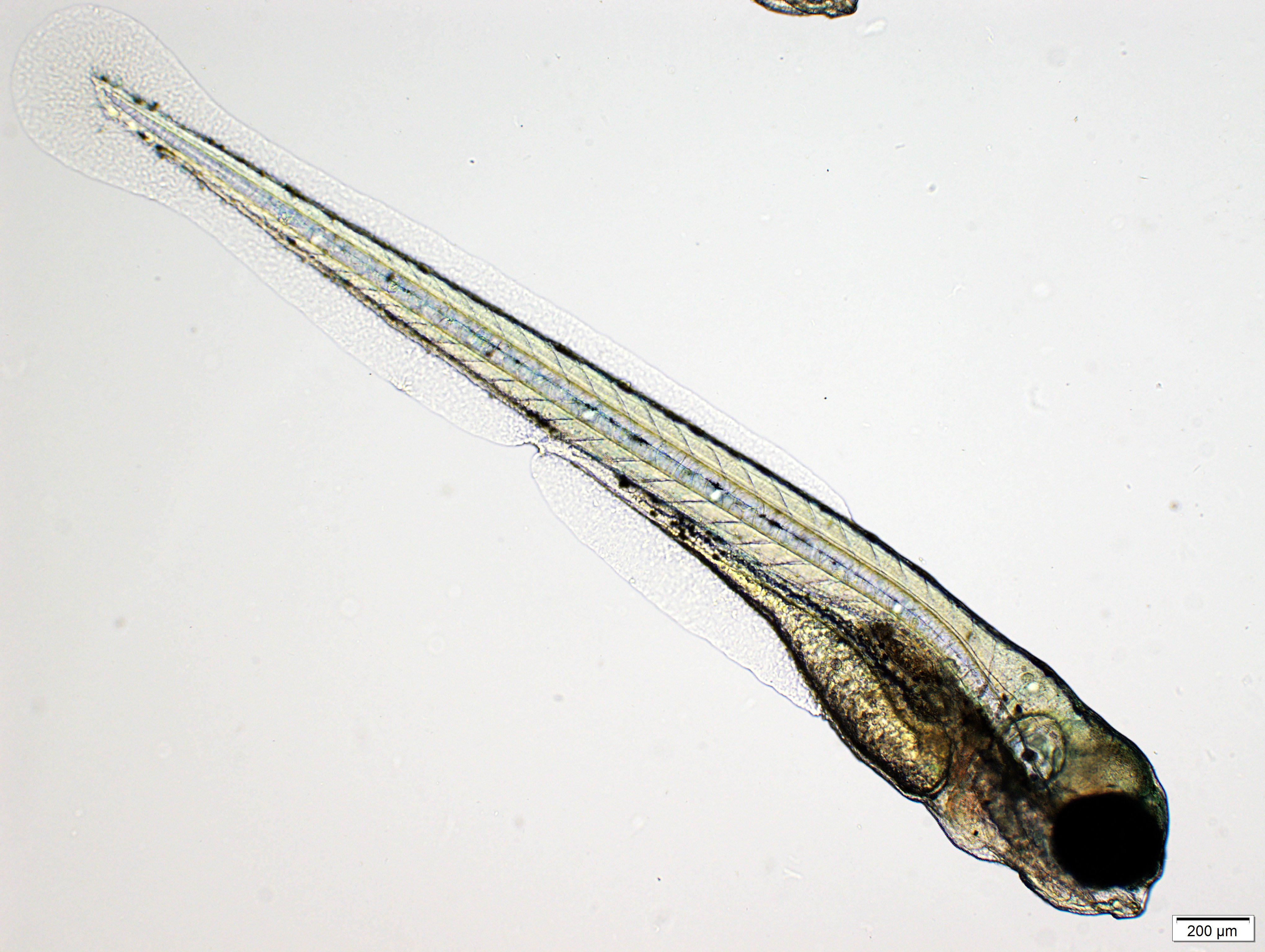

Supplement: Supplementary file 4 — Source data Fig. 2 [file 44321_2025_204_MOESM4_ESM.zip › Figure 2/2D/2D-lateral view.jpg]

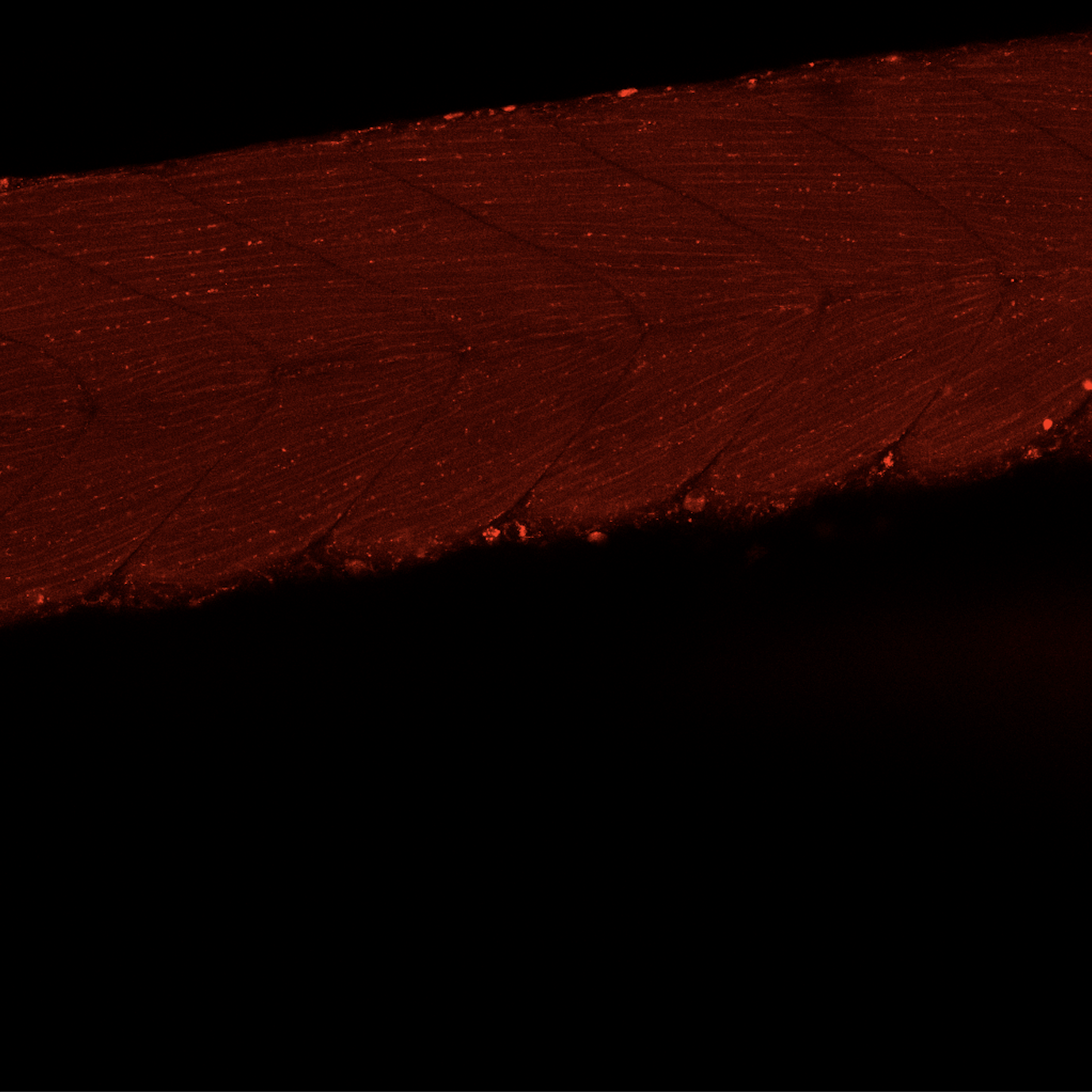

Supplement: Supplementary file 5 — Source data Fig. 3 [file 44321_2025_204_MOESM5_ESM.zip › Figure 3 Source Data/3A-C/Figure 3A.png]

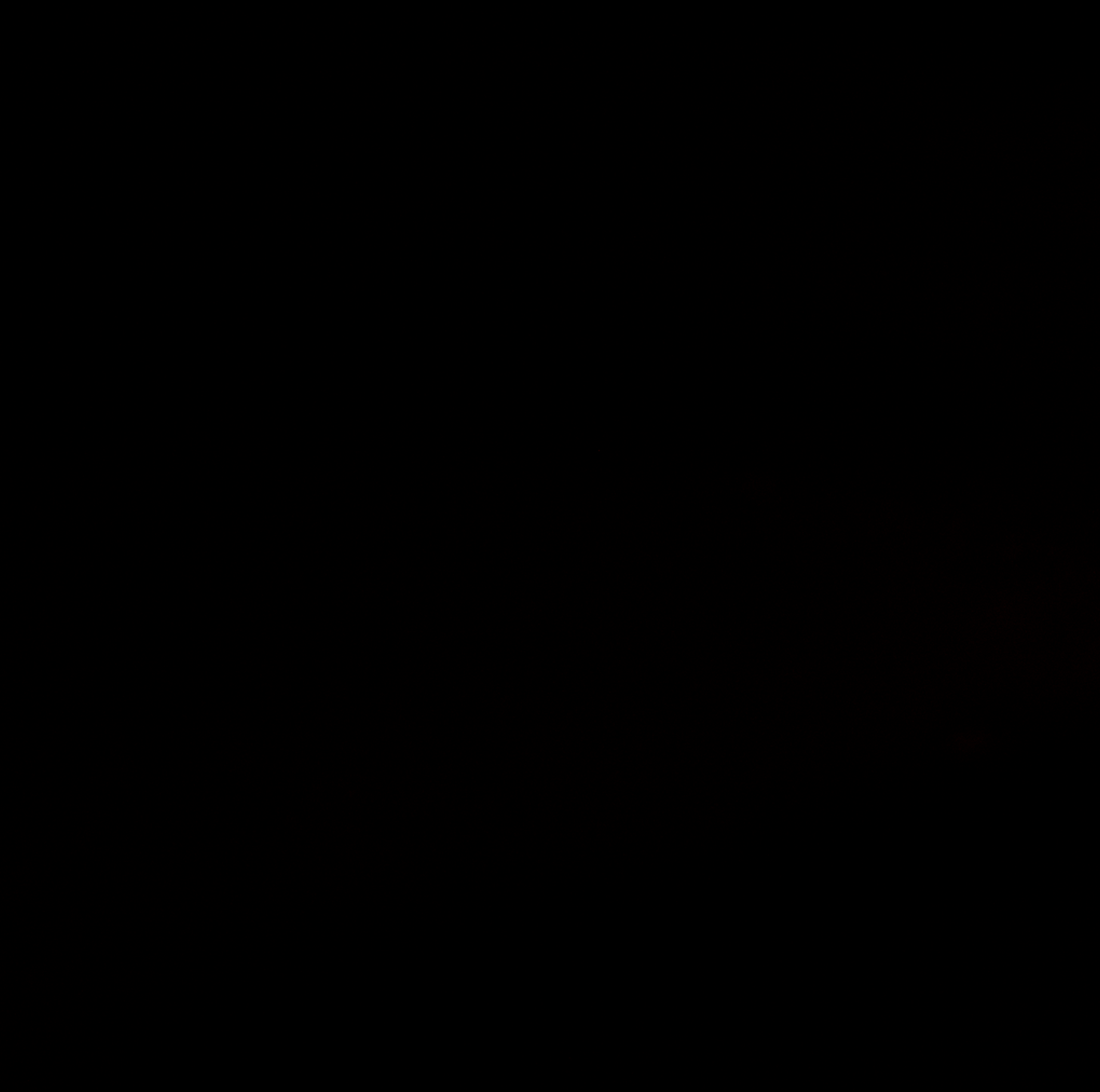

Supplement: Supplementary file 5 — Source data Fig. 3 [file 44321_2025_204_MOESM5_ESM.zip › Figure 3 Source Data/3A-C/Figure 3B.png]

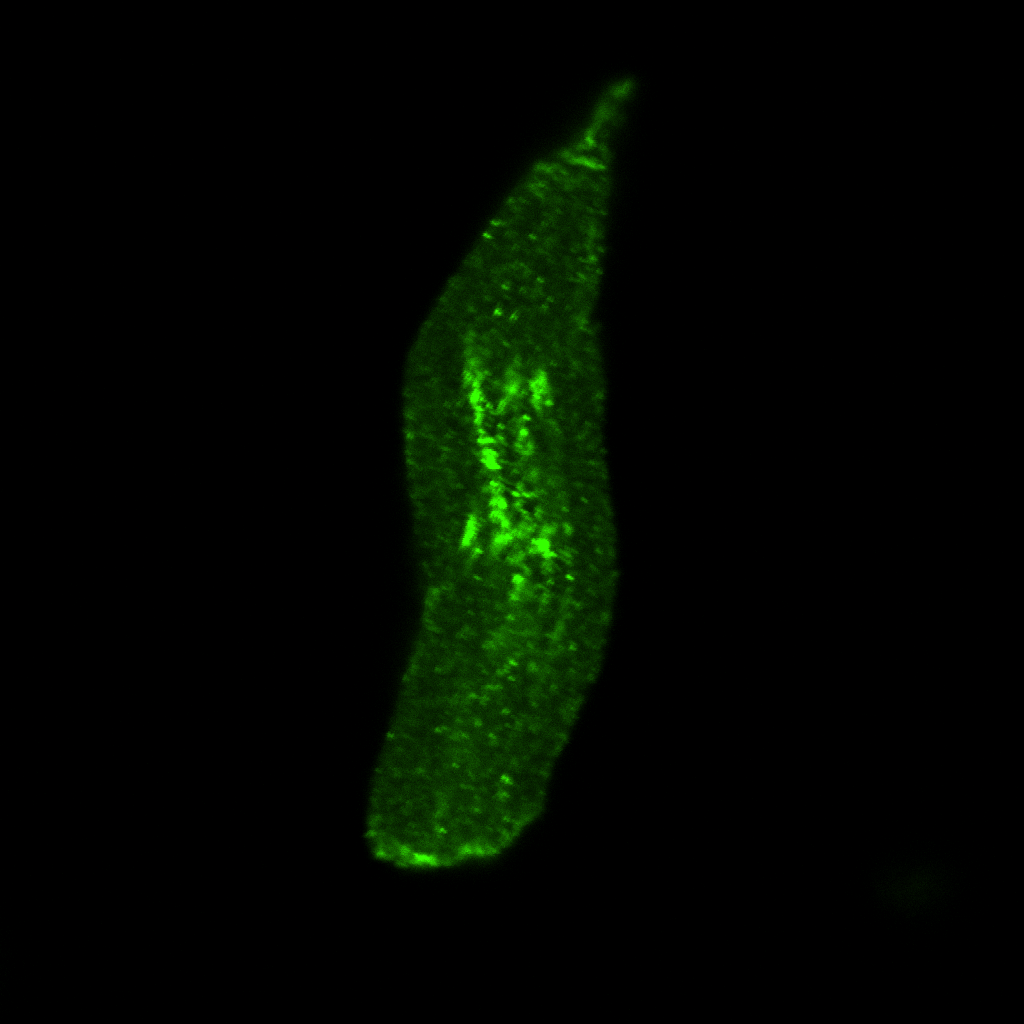

Supplement: Supplementary file 5 — Source data Fig. 3 [file 44321_2025_204_MOESM5_ESM.zip › Figure 3 Source Data/3F/3F-single plane confocal image of Lamp1 staining in WT.tif]

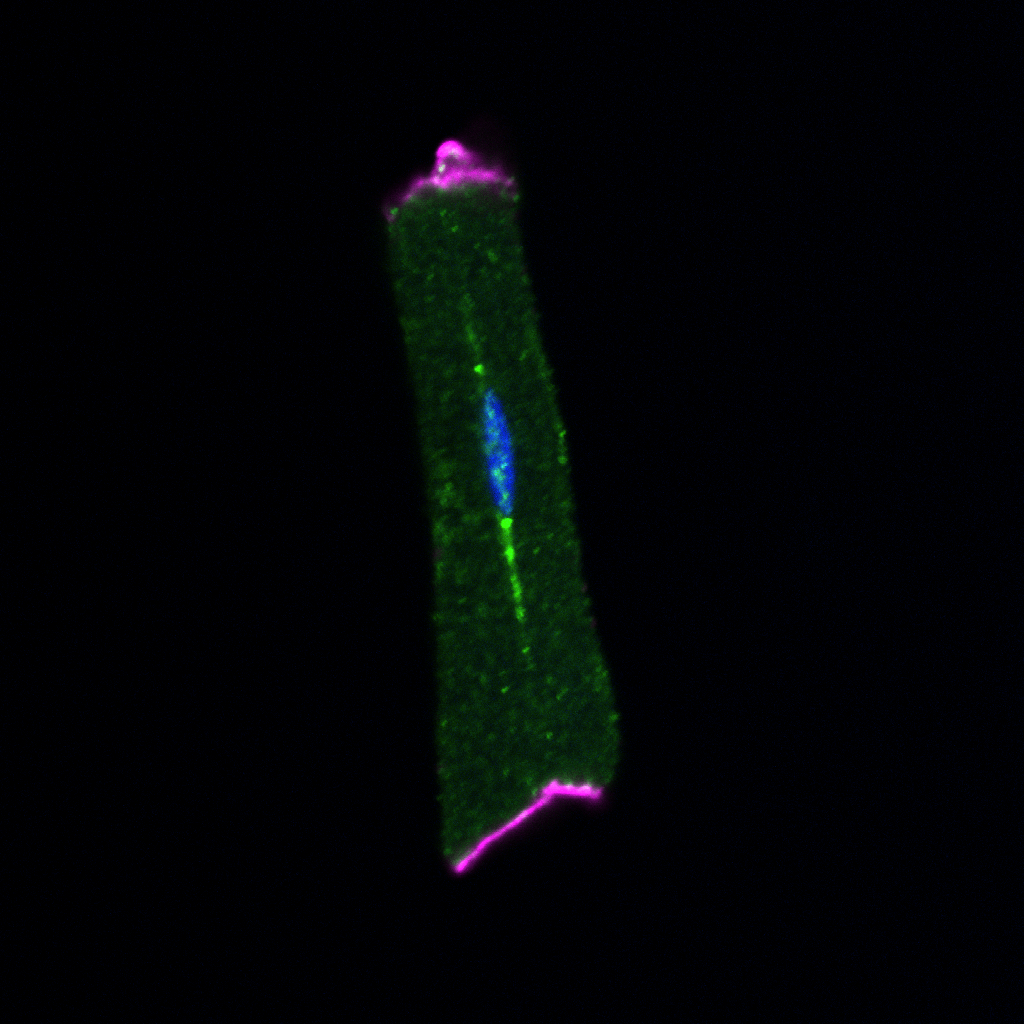

Supplement: Supplementary file 5 — Source data Fig. 3 [file 44321_2025_204_MOESM5_ESM.zip › Figure 3 Source Data/3F/3F-single plane confocal image of Lamp1, dys, and dapi staining in vma21 mutant.tif]

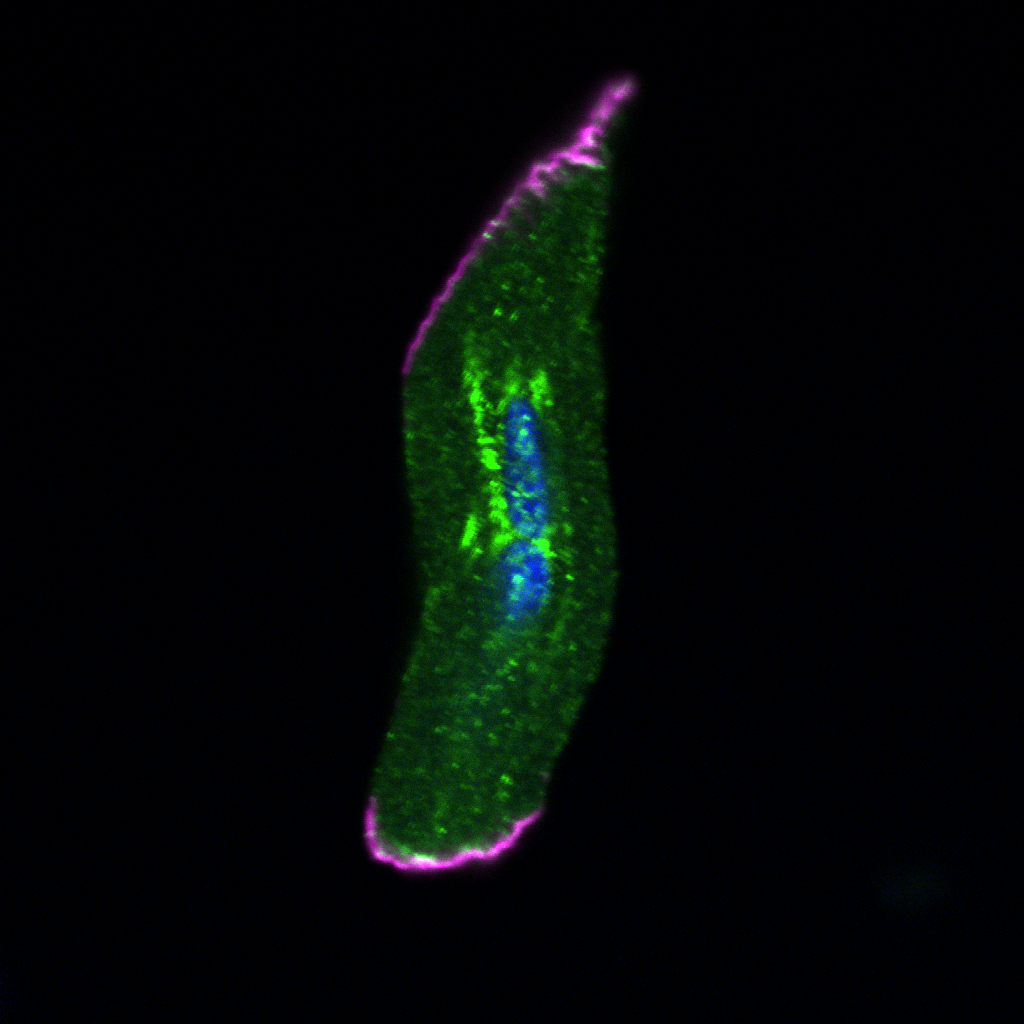

Supplement: Supplementary file 5 — Source data Fig. 3 [file 44321_2025_204_MOESM5_ESM.zip › Figure 3 Source Data/3F/3F- single plane confocal image of Lamp1, dys, and dapi staining in WT.tif]

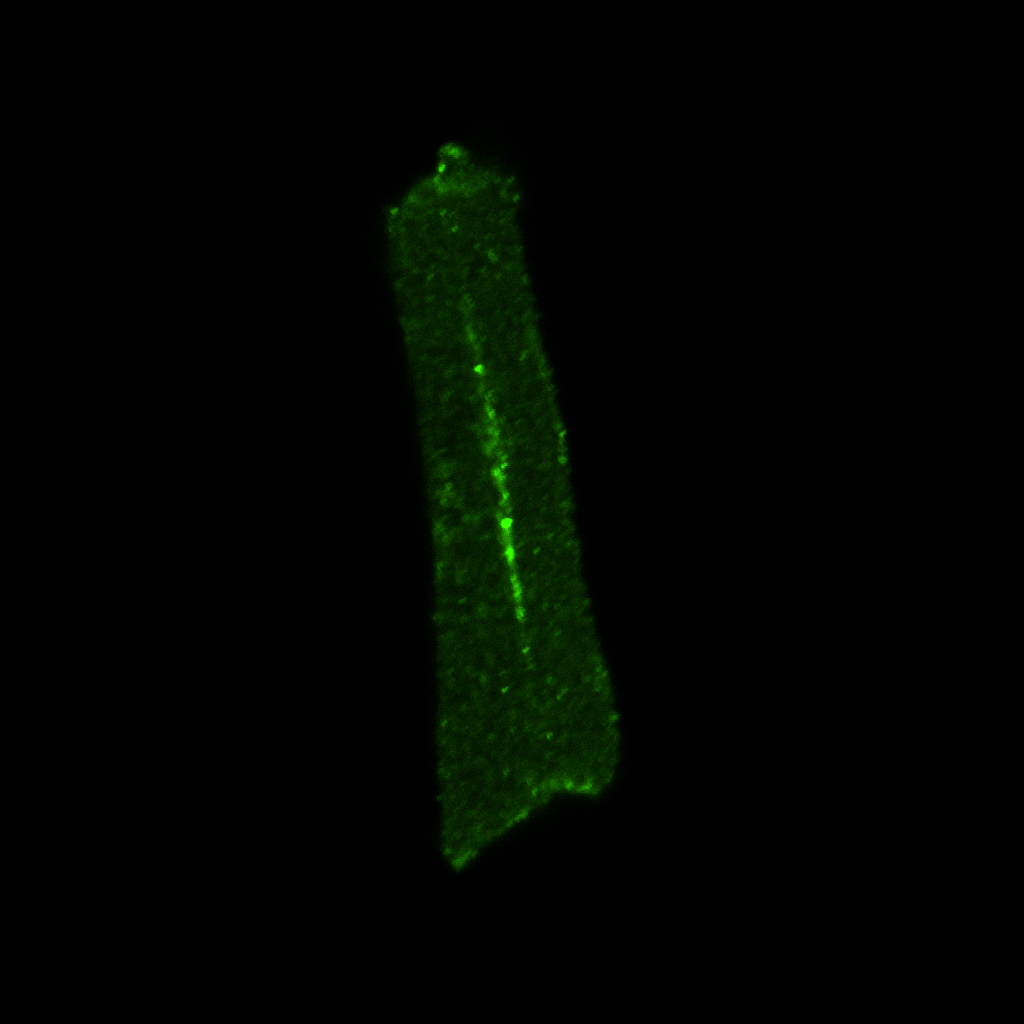

Supplement: Supplementary file 5 — Source data Fig. 3 [file 44321_2025_204_MOESM5_ESM.zip › Figure 3 Source Data/3F/3F-single plane confocal image of Lamp1 staining in vma21 mutant.tif]

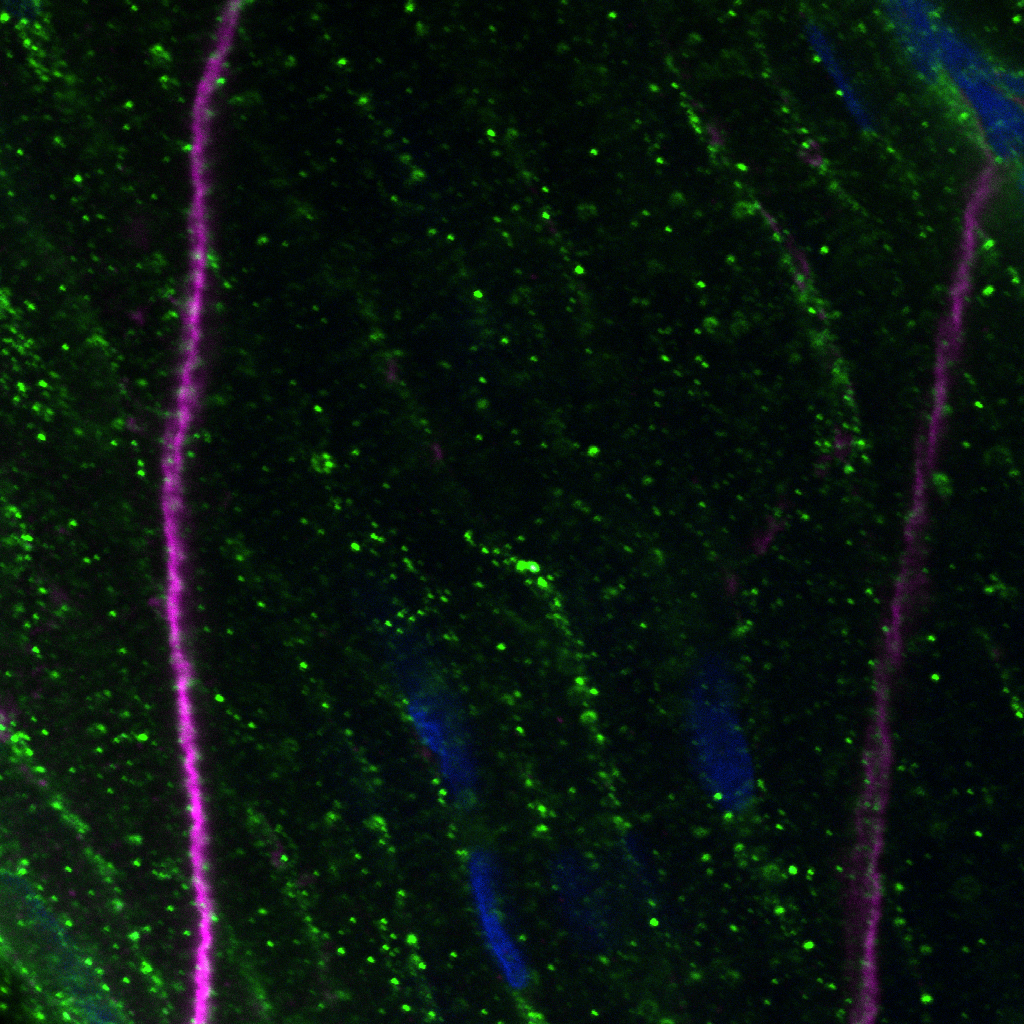

Supplement: Supplementary file 5 — Source data Fig. 3 [file 44321_2025_204_MOESM5_ESM.zip › Figure 3 Source Data/3D-E/3E/3E- Whole-mount confocal image of Lamp1, Dys, and dapi staining in vma21 mutant.tif]

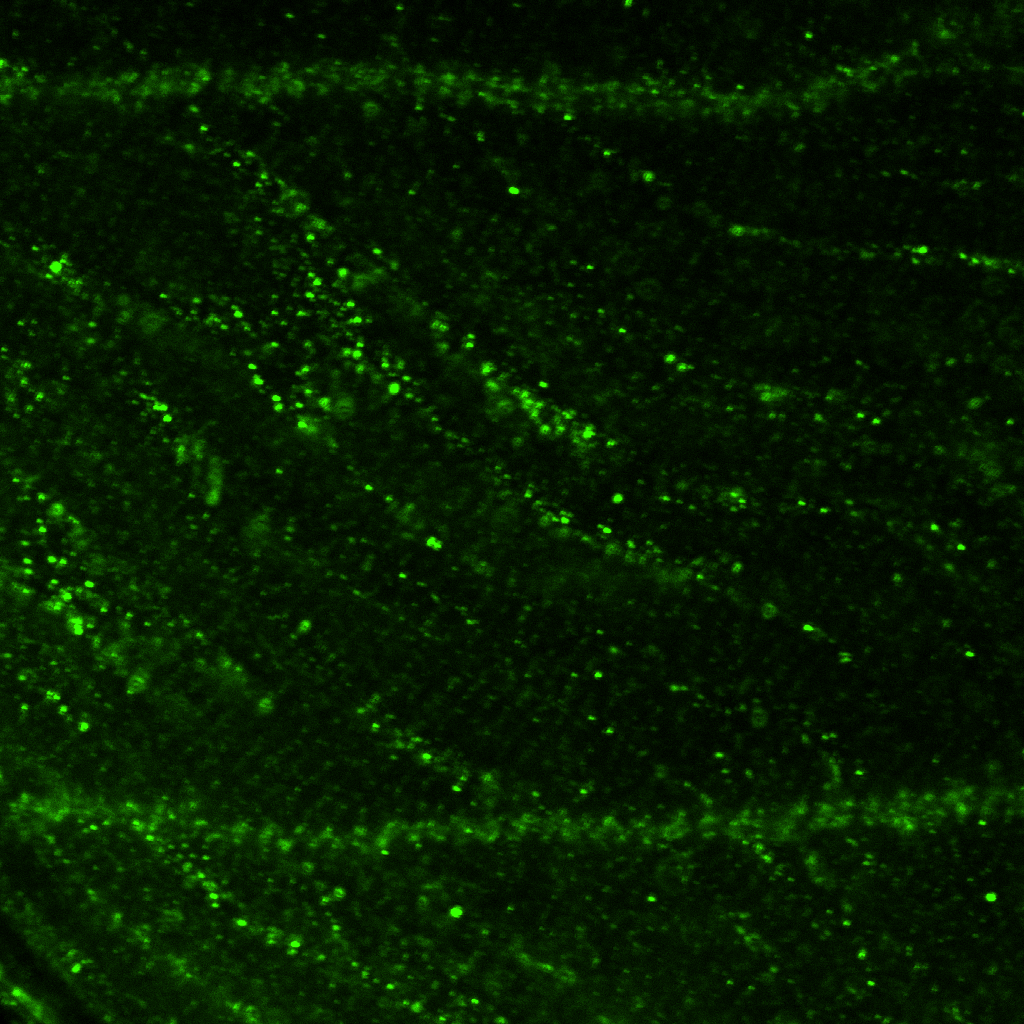

Supplement: Supplementary file 5 — Source data Fig. 3 [file 44321_2025_204_MOESM5_ESM.zip › Figure 3 Source Data/3D-E/3E/3E- Whole-mount confocal image of Lamp1 staining in WT.tif]

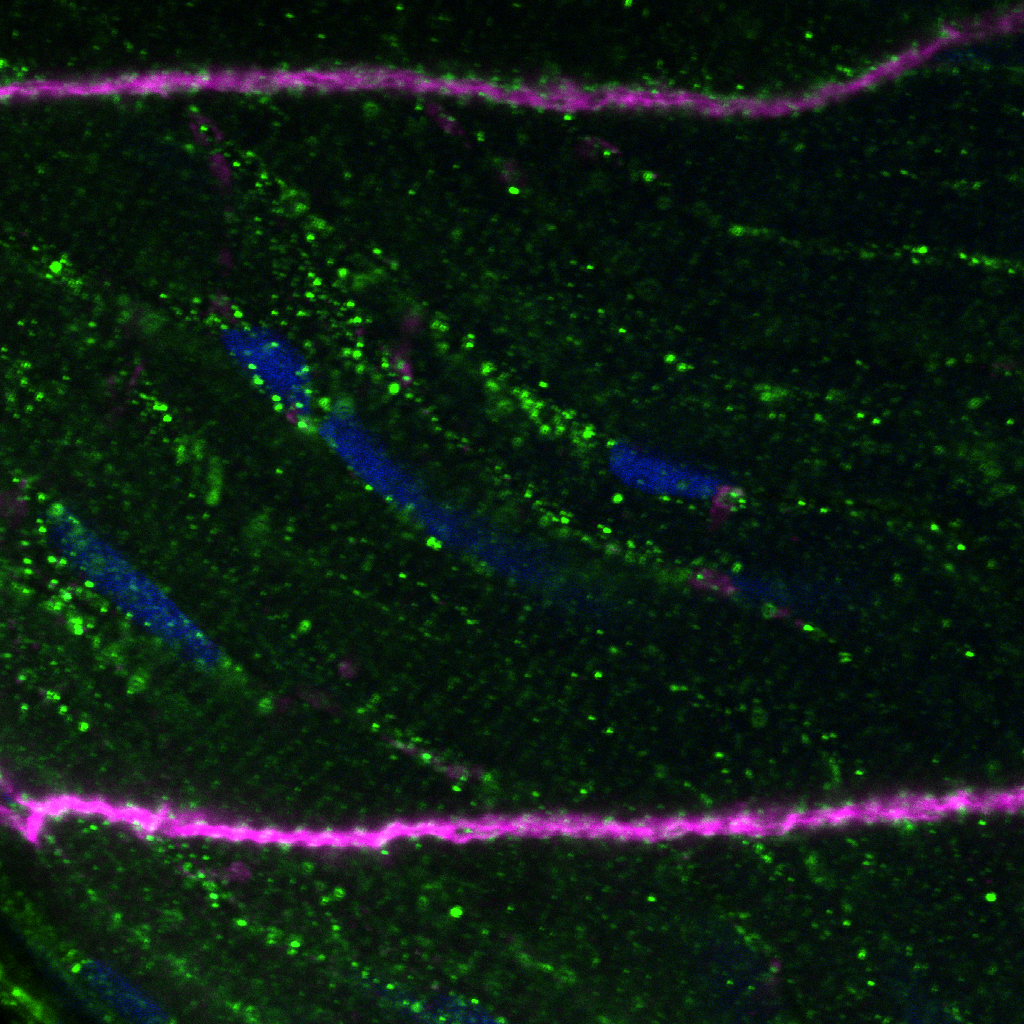

Supplement: Supplementary file 5 — Source data Fig. 3 [file 44321_2025_204_MOESM5_ESM.zip › Figure 3 Source Data/3D-E/3E/3E- Whole-mount confocal image of Lamp1, Dys, and dapi staining in WT.tif]

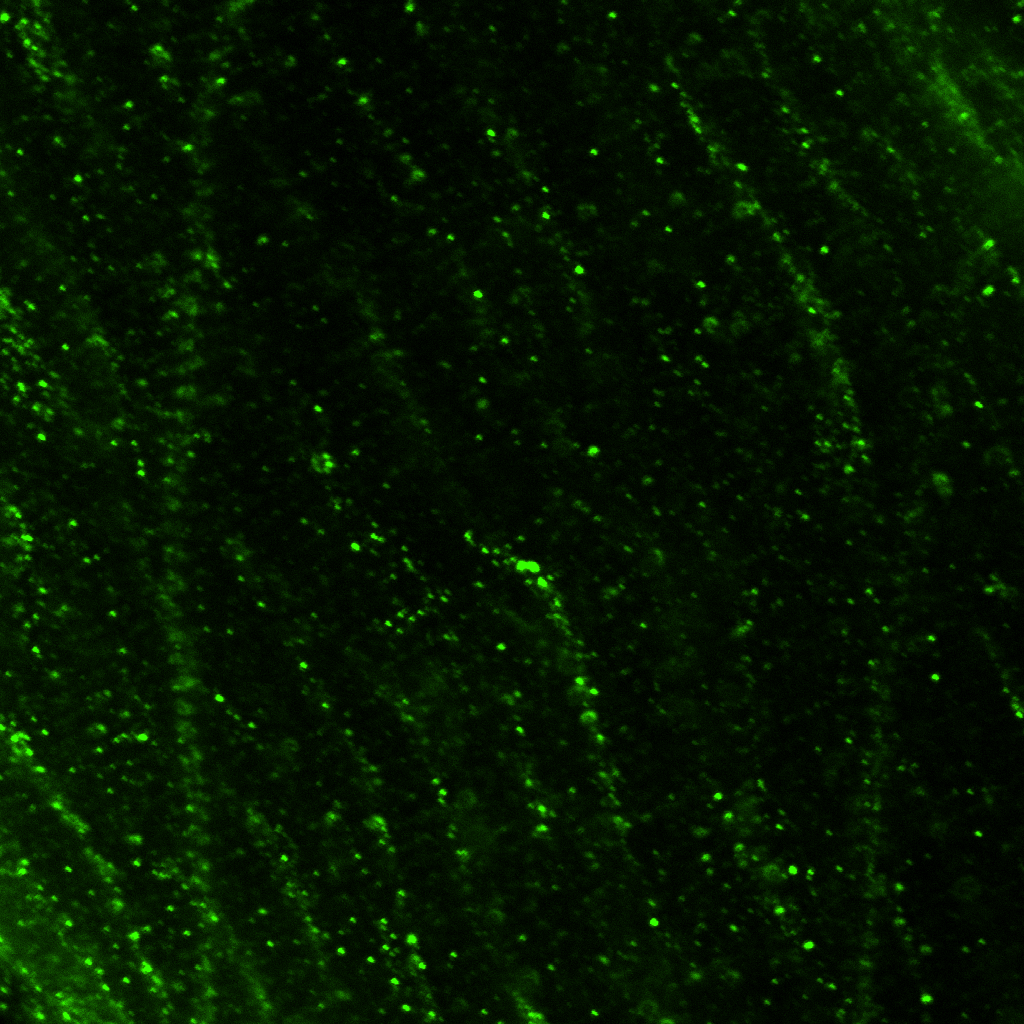

Supplement: Supplementary file 5 — Source data Fig. 3 [file 44321_2025_204_MOESM5_ESM.zip › Figure 3 Source Data/3D-E/3E/3E- Whole-mount confocal image of Lamp1 staining in vma21 mutant.tif]

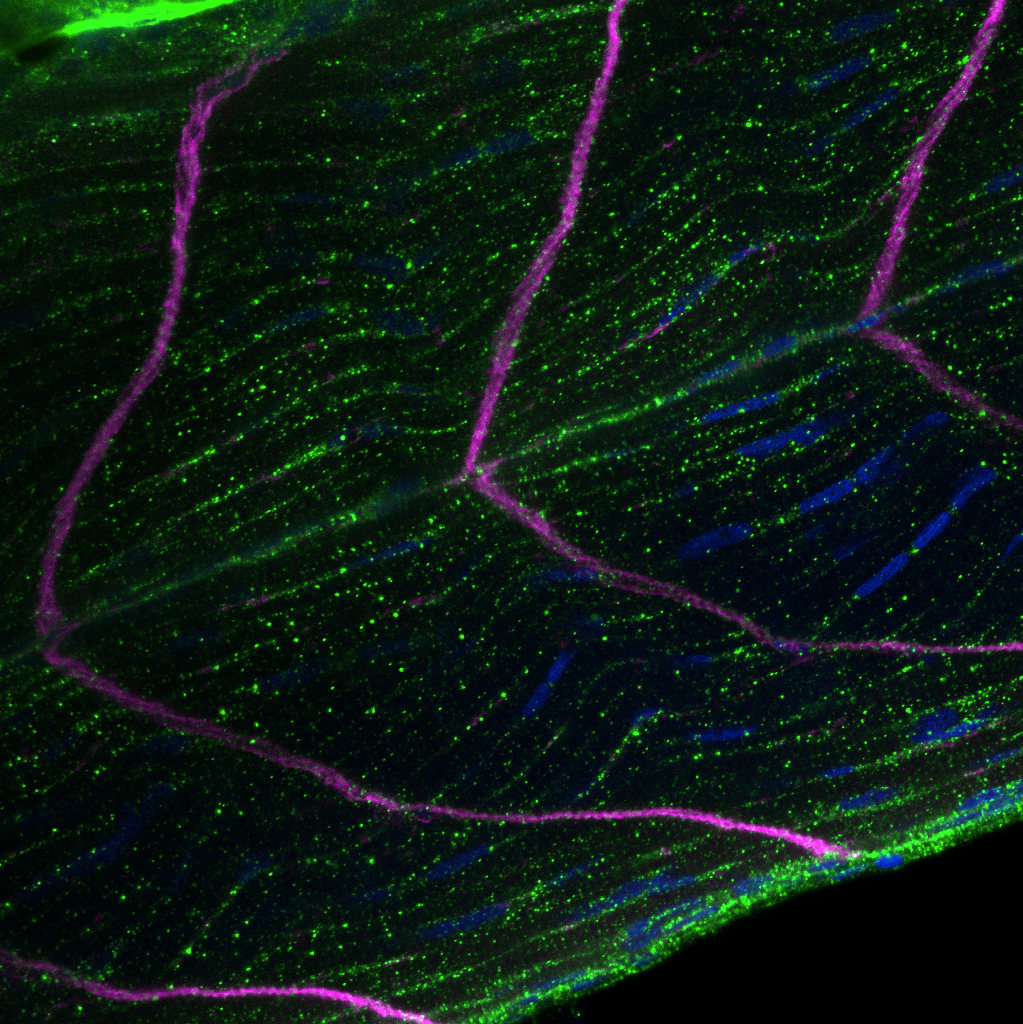

Supplement: Supplementary file 5 — Source data Fig. 3 [file 44321_2025_204_MOESM5_ESM.zip › Figure 3 Source Data/3D-E/3D/3D- Z-stack of whole-mount Lamp1, dys, and dapi staining in vma21 mutant.tif]

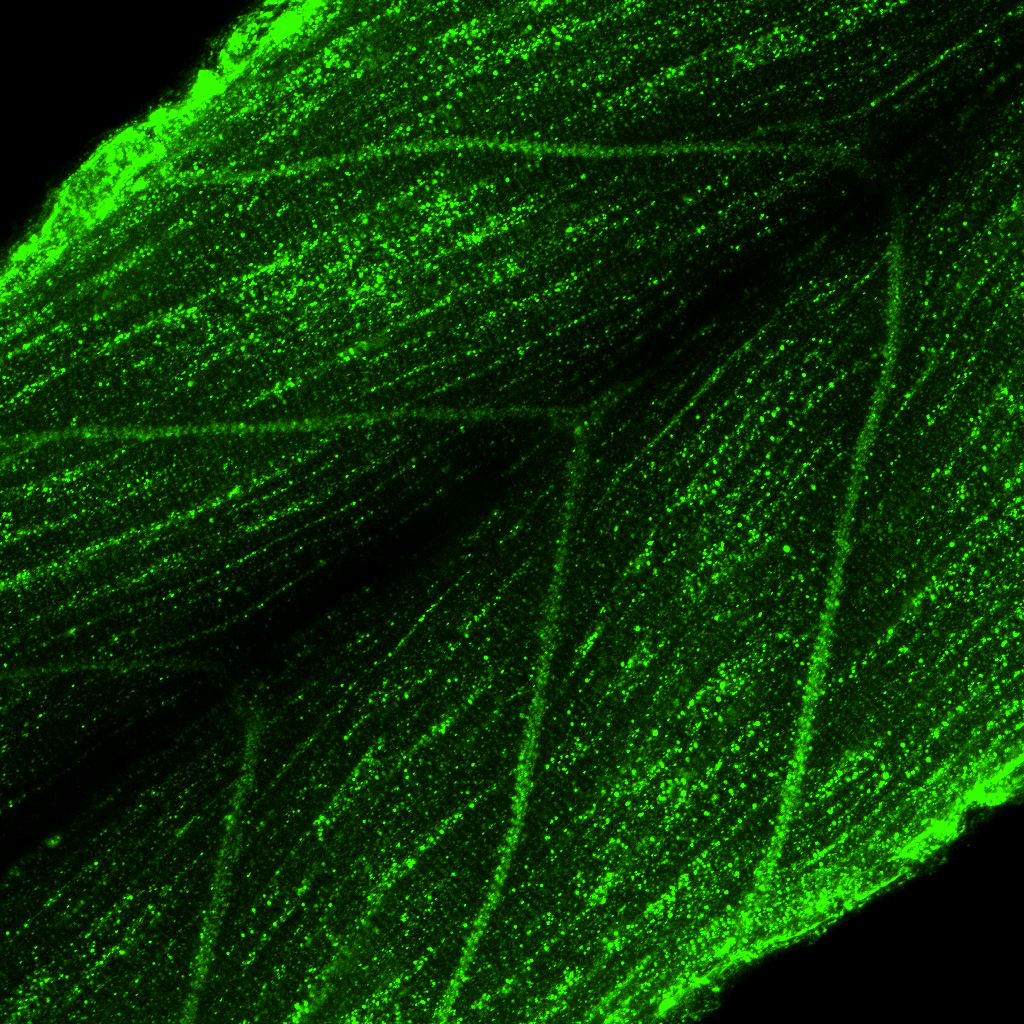

Supplement: Supplementary file 5 — Source data Fig. 3 [file 44321_2025_204_MOESM5_ESM.zip › Figure 3 Source Data/3D-E/3D/3D- Z-stack of whole-mount Lamp1 staining in WT.tif]

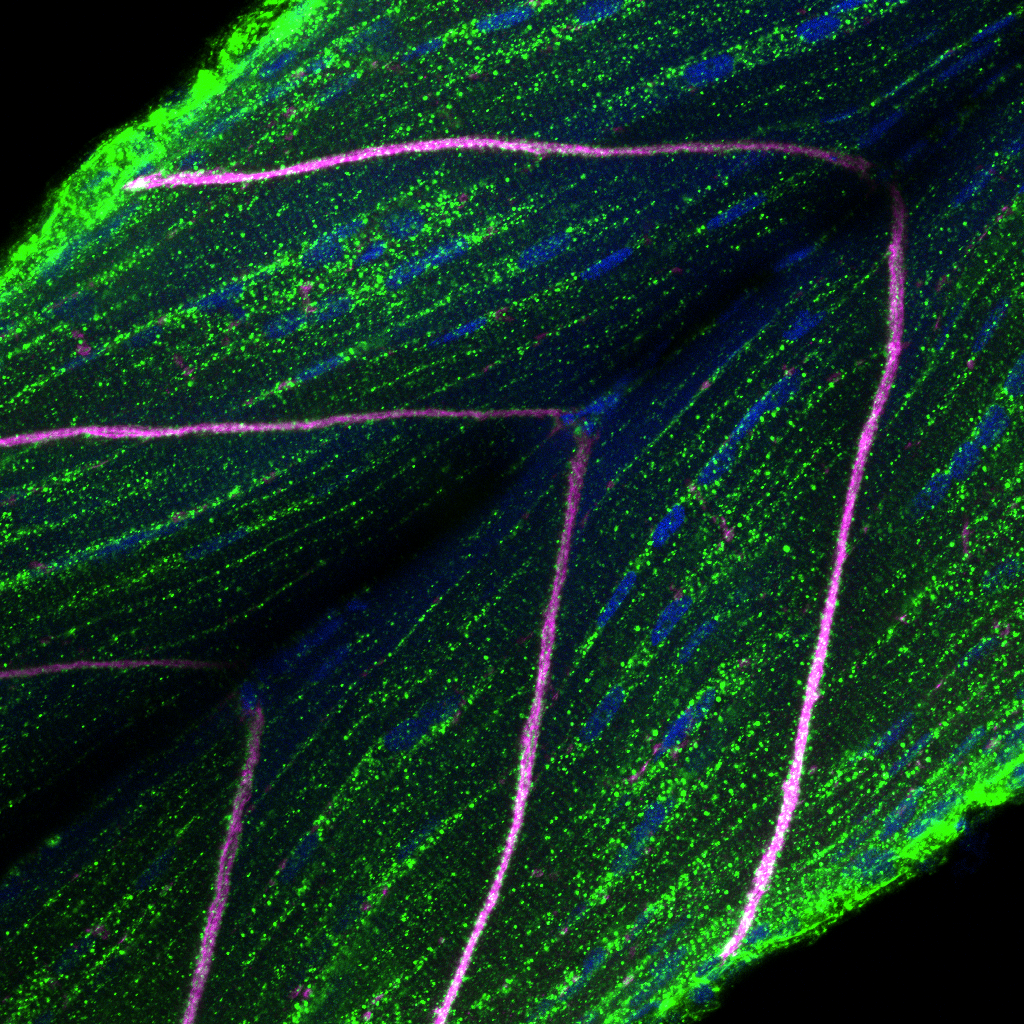

Supplement: Supplementary file 5 — Source data Fig. 3 [file 44321_2025_204_MOESM5_ESM.zip › Figure 3 Source Data/3D-E/3D/3D- Z-stack of whole-mount Lamp1, dys, and dapi staining in WT.tif]

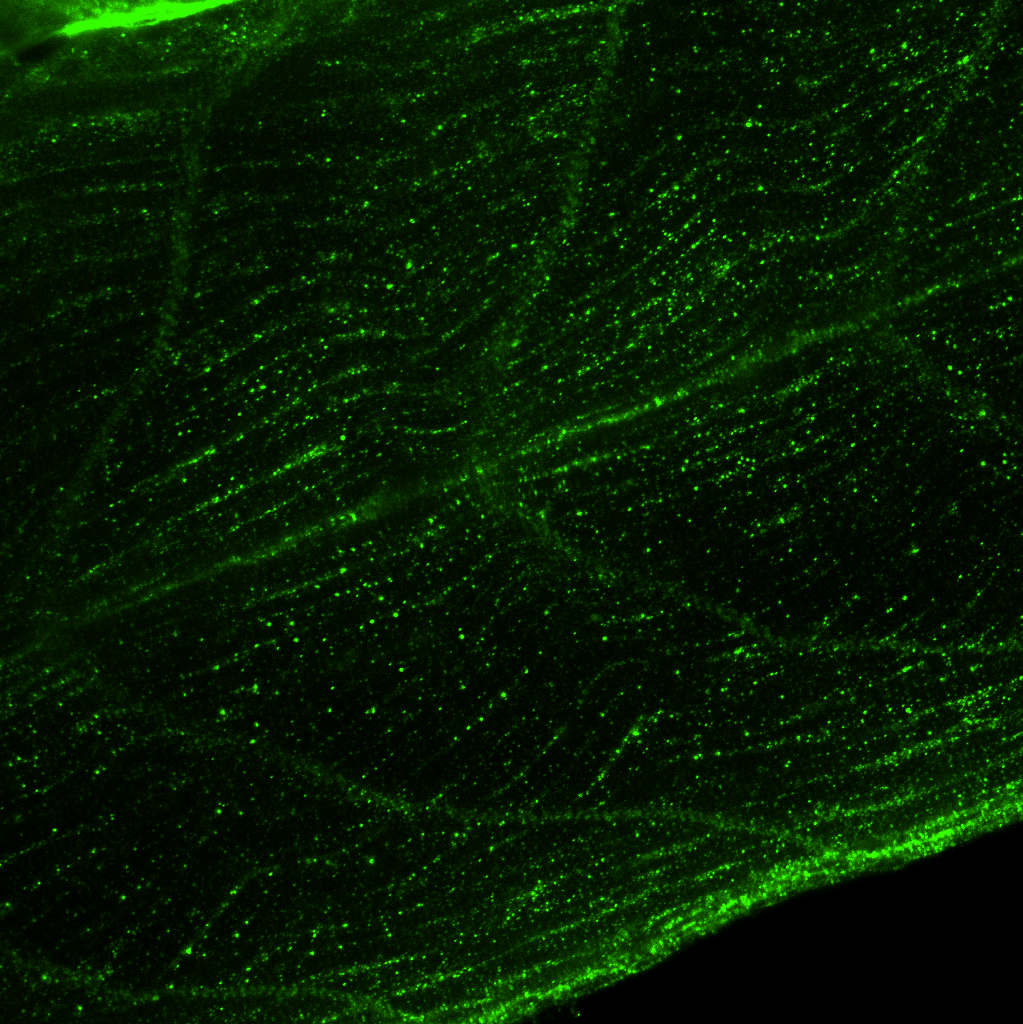

Supplement: Supplementary file 5 — Source data Fig. 3 [file 44321_2025_204_MOESM5_ESM.zip › Figure 3 Source Data/3D-E/3D/3D- Z-stack of whole-mount Lamp1 staining in vma21 mutant.tif]

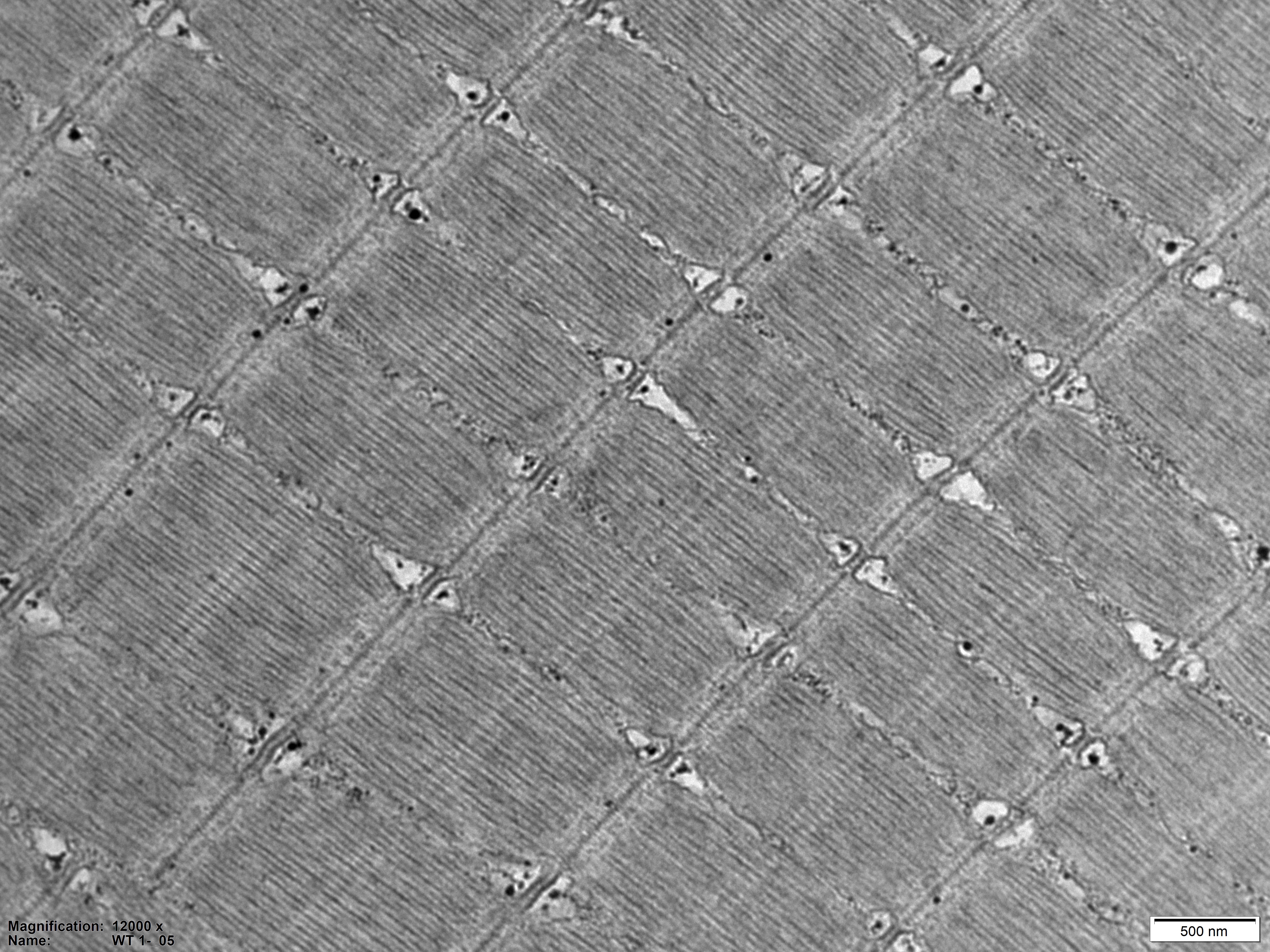

Supplement: Supplementary file 6 — Source data Fig. 4 [file 44321_2025_204_MOESM6_ESM.zip › Figure 4/4A-B/4A- WT EM.jpg]

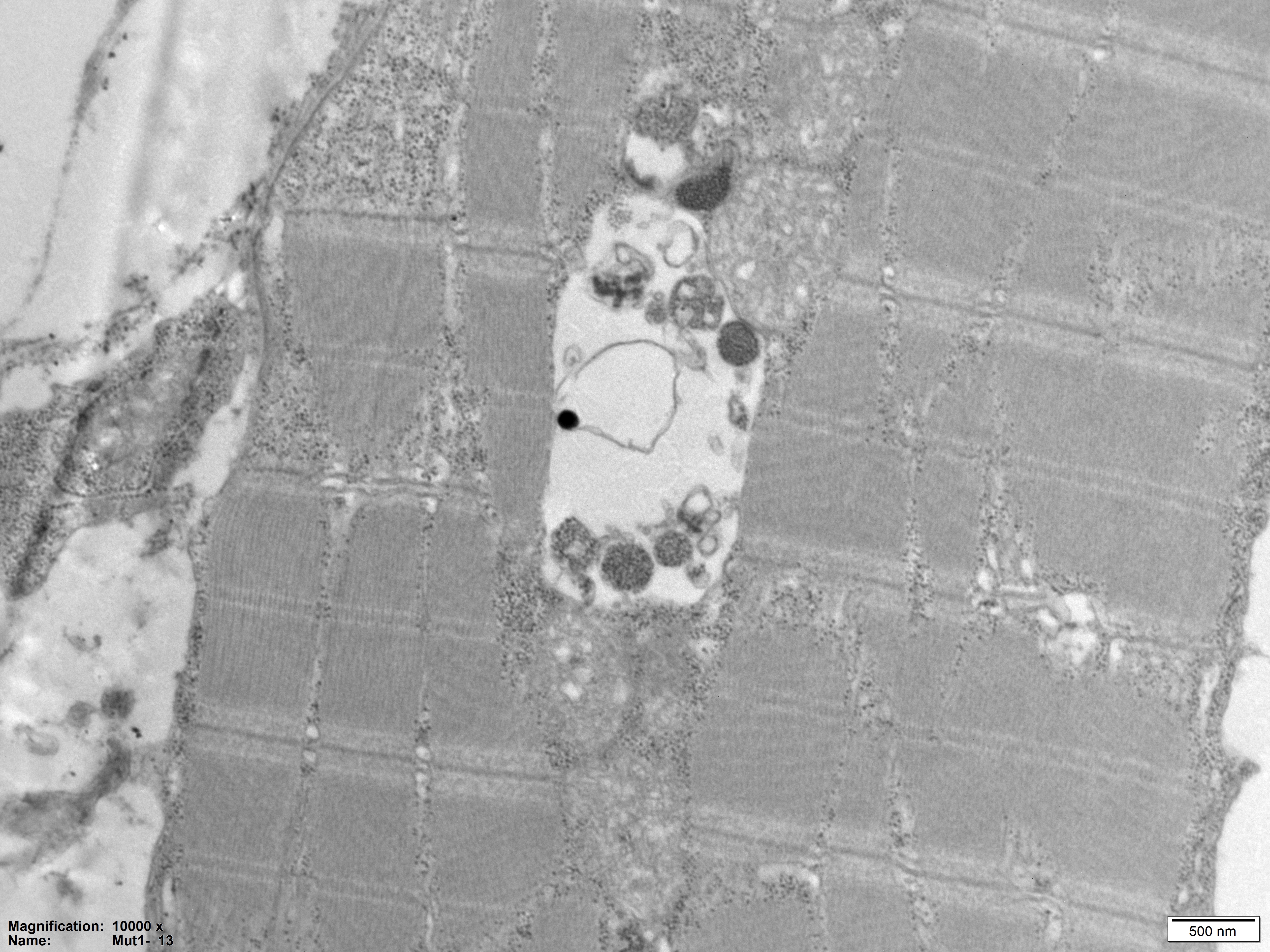

Supplement: Supplementary file 6 — Source data Fig. 4 [file 44321_2025_204_MOESM6_ESM.zip › Figure 4/4A-B/4B- Mutant EM.jpg]

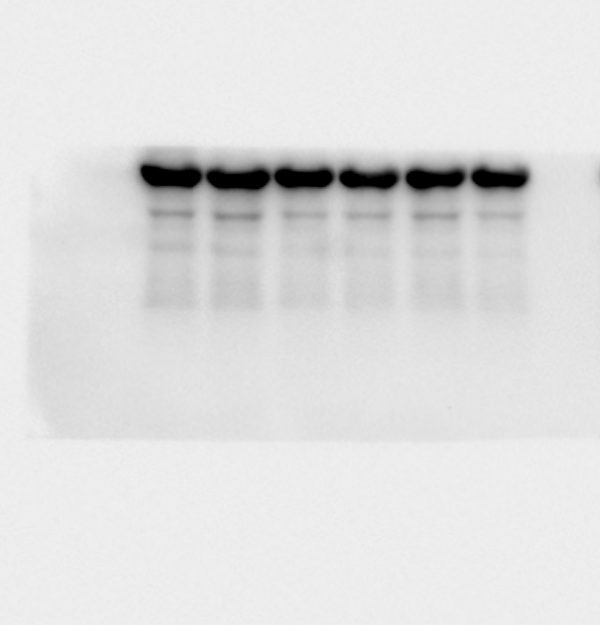

Supplement: Supplementary file 6 — Source data Fig. 4 [file 44321_2025_204_MOESM6_ESM.zip › Figure 4/4C-D/4C- b actin blot.tif]

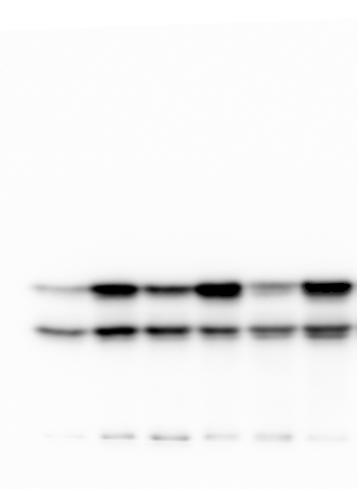

Supplement: Supplementary file 6 — Source data Fig. 4 [file 44321_2025_204_MOESM6_ESM.zip › Figure 4/4C-D/4C- LC3 blot.tif]

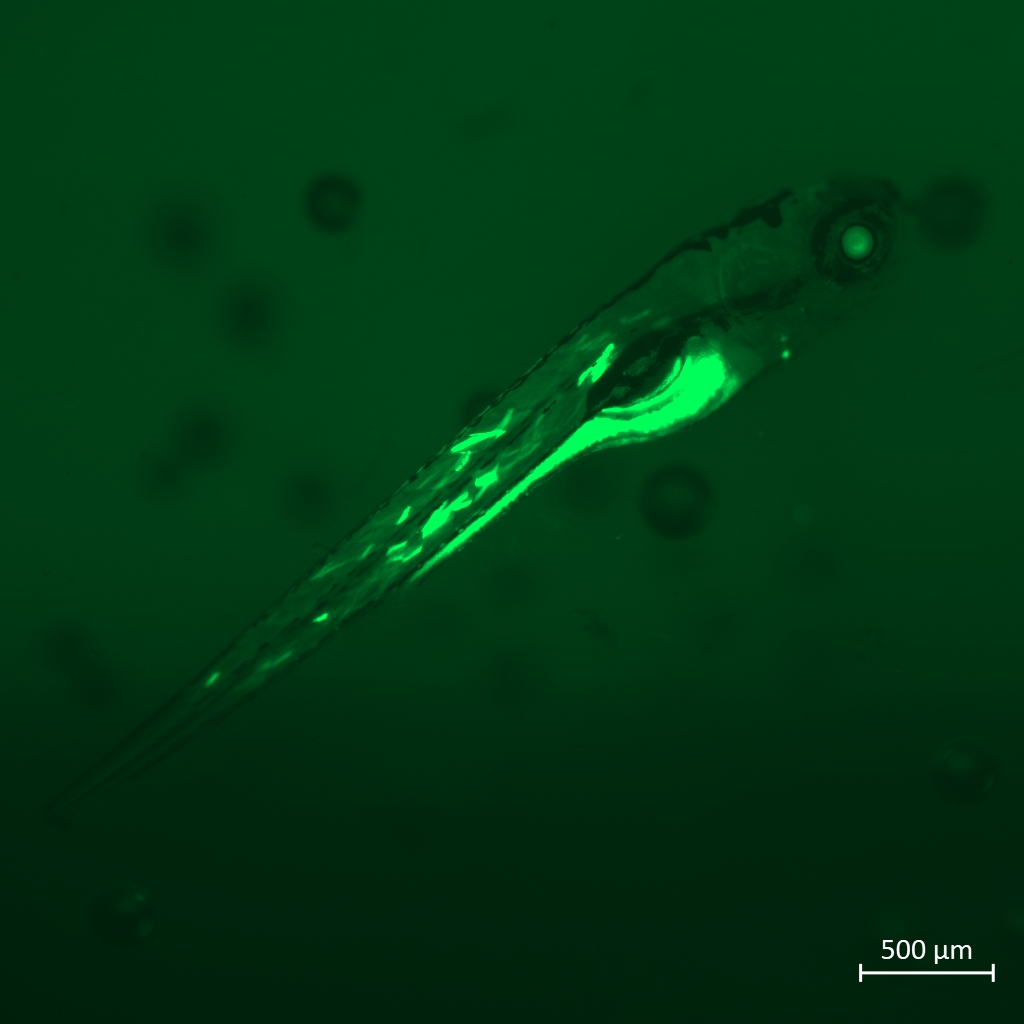

Supplement: Supplementary file 6 — Source data Fig. 4 [file 44321_2025_204_MOESM6_ESM.zip › Figure 4/4E-I/4E.jpg]

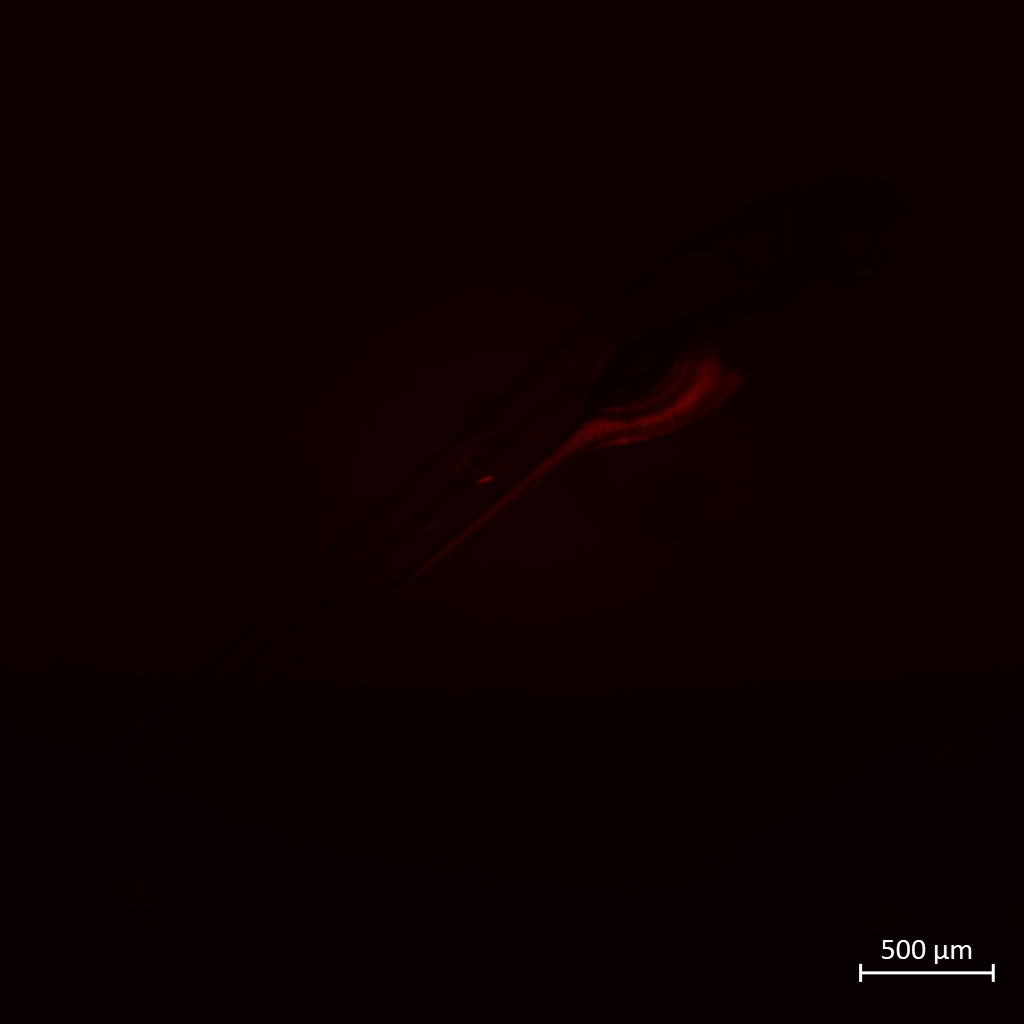

Supplement: Supplementary file 6 — Source data Fig. 4 [file 44321_2025_204_MOESM6_ESM.zip › Figure 4/4E-I/4F.jpg]

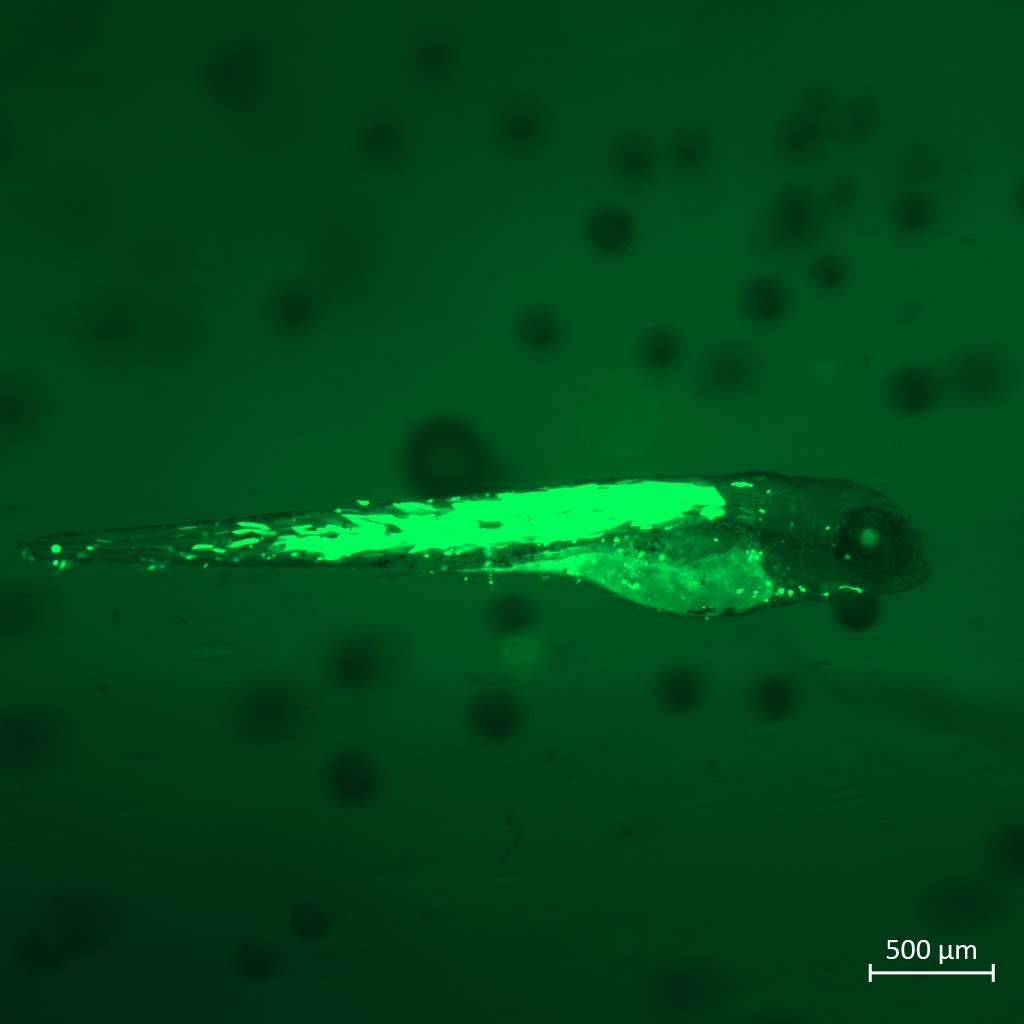

Supplement: Supplementary file 6 — Source data Fig. 4 [file 44321_2025_204_MOESM6_ESM.zip › Figure 4/4E-I/4G.jpg]

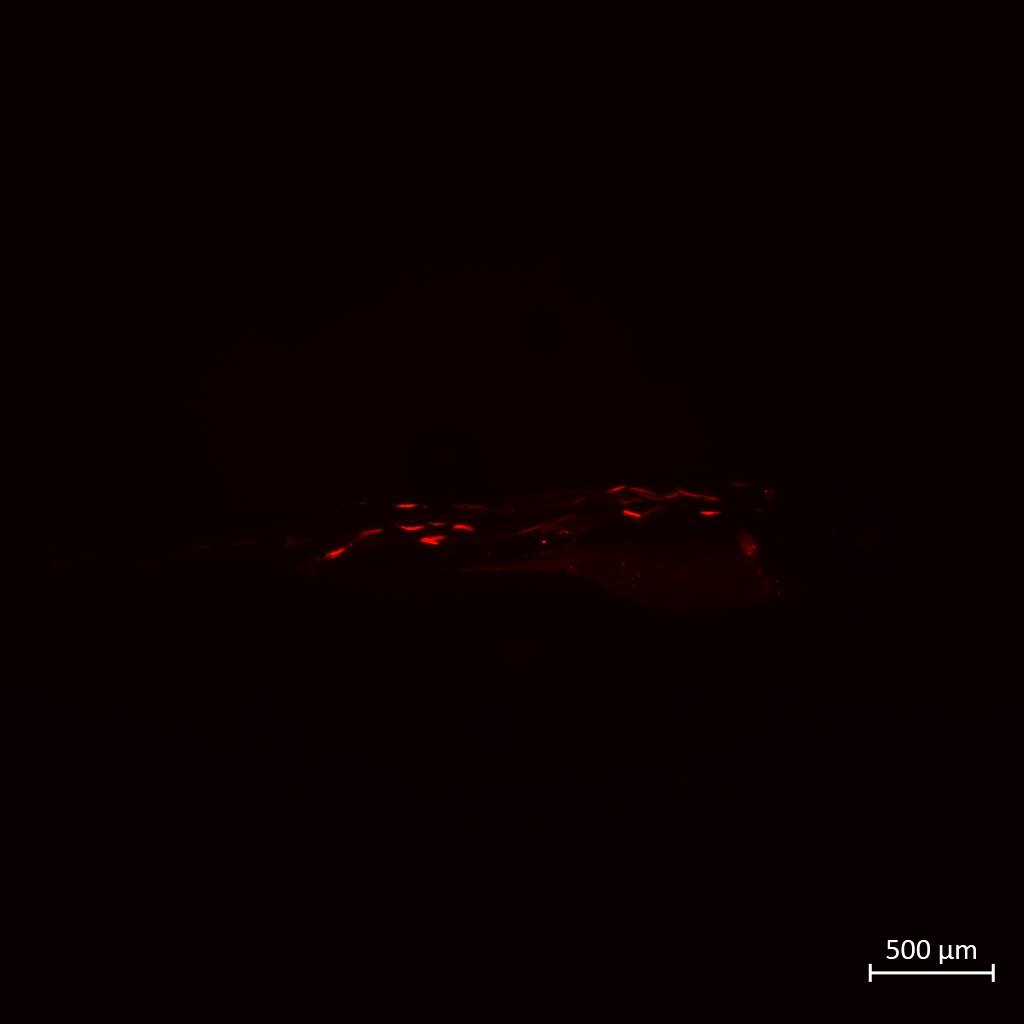

Supplement: Supplementary file 6 — Source data Fig. 4 [file 44321_2025_204_MOESM6_ESM.zip › Figure 4/4E-I/4H.jpg]

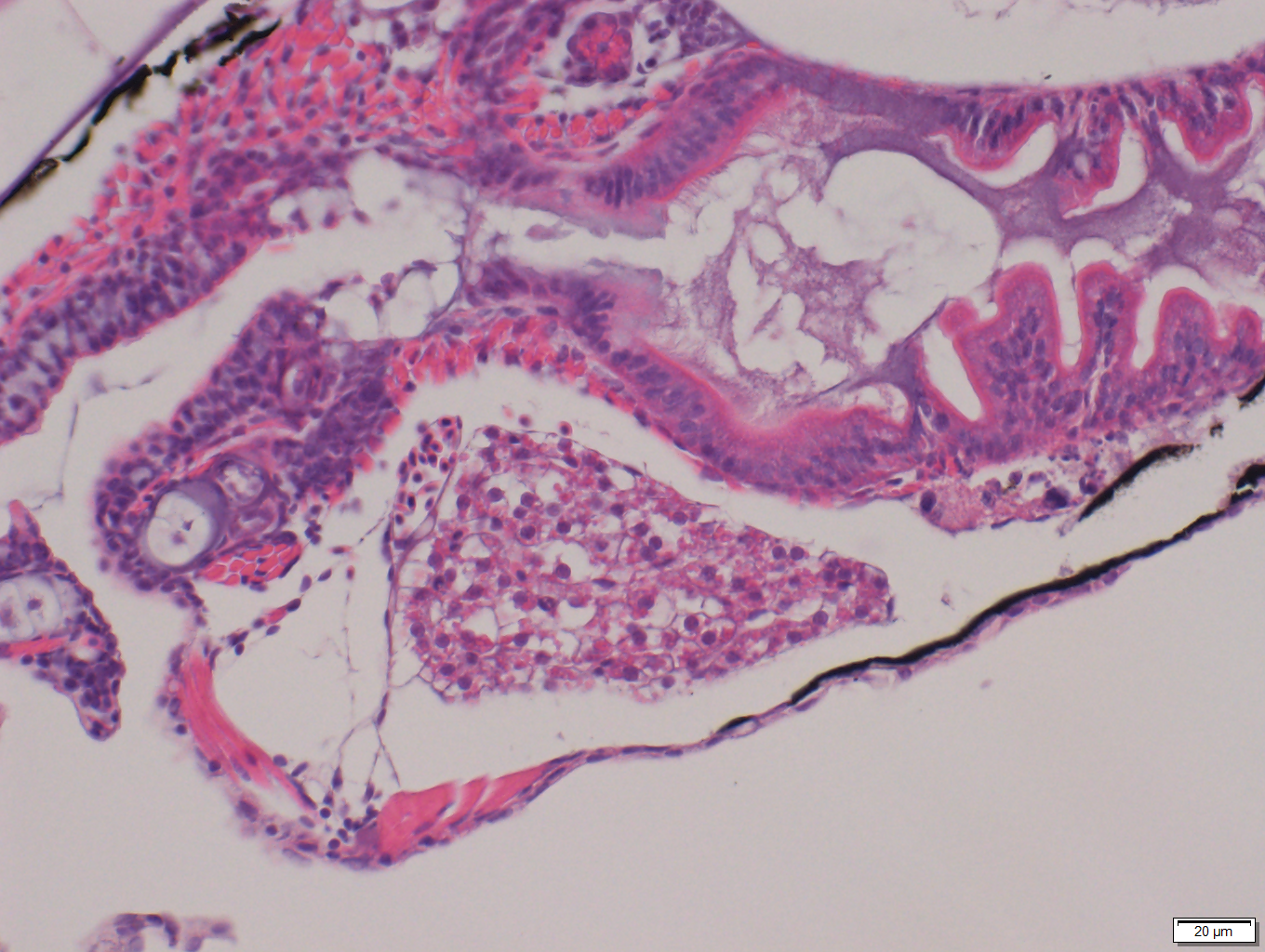

Supplement: Supplementary file 7 — Source data Fig. 5 [file 44321_2025_204_MOESM7_ESM.zip › Figure 5/5A.tif]

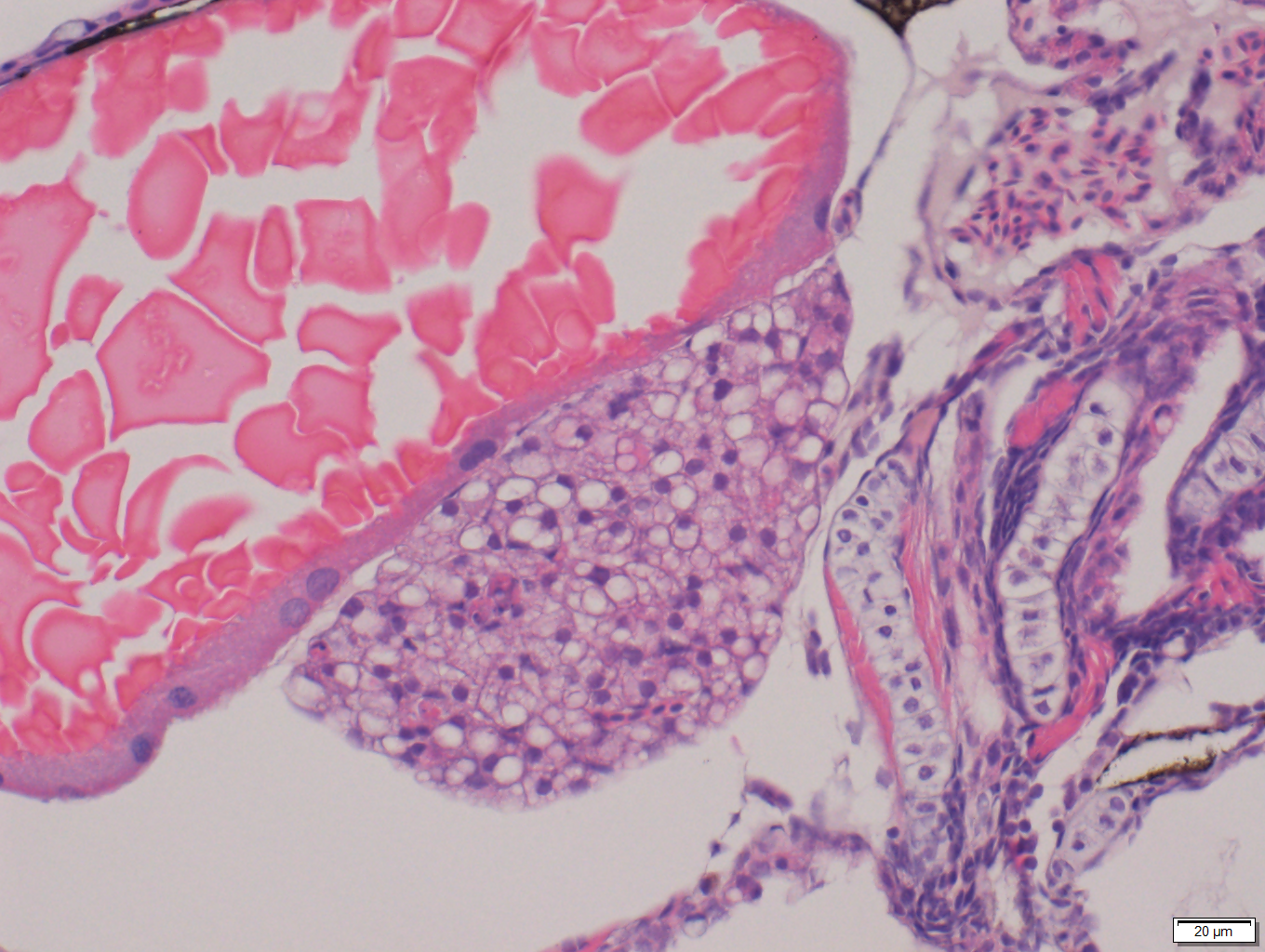

Supplement: Supplementary file 7 — Source data Fig. 5 [file 44321_2025_204_MOESM7_ESM.zip › Figure 5/5B.tif]

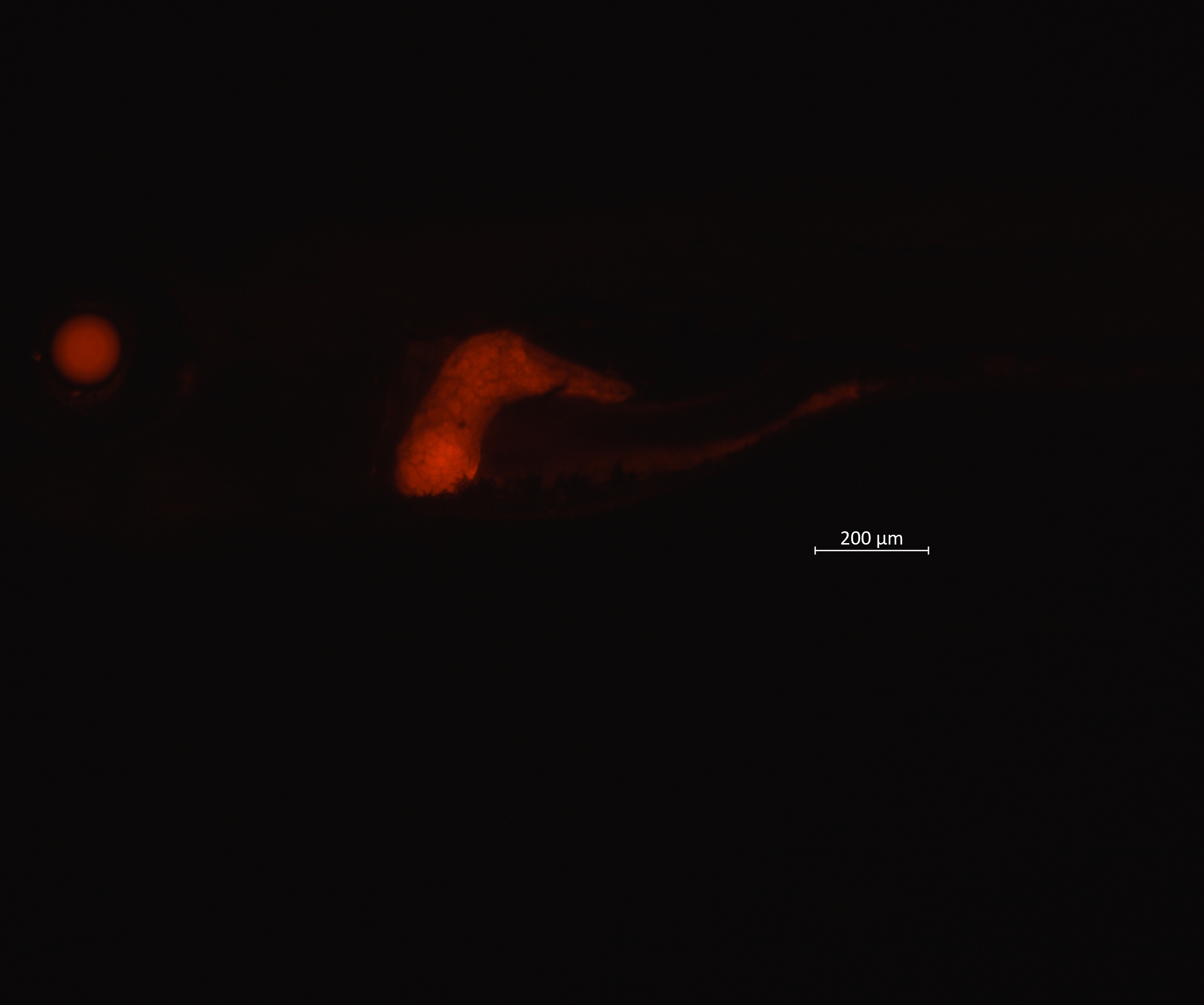

Supplement: Supplementary file 7 — Source data Fig. 5 [file 44321_2025_204_MOESM7_ESM.zip › Figure 5/5C.jpg]

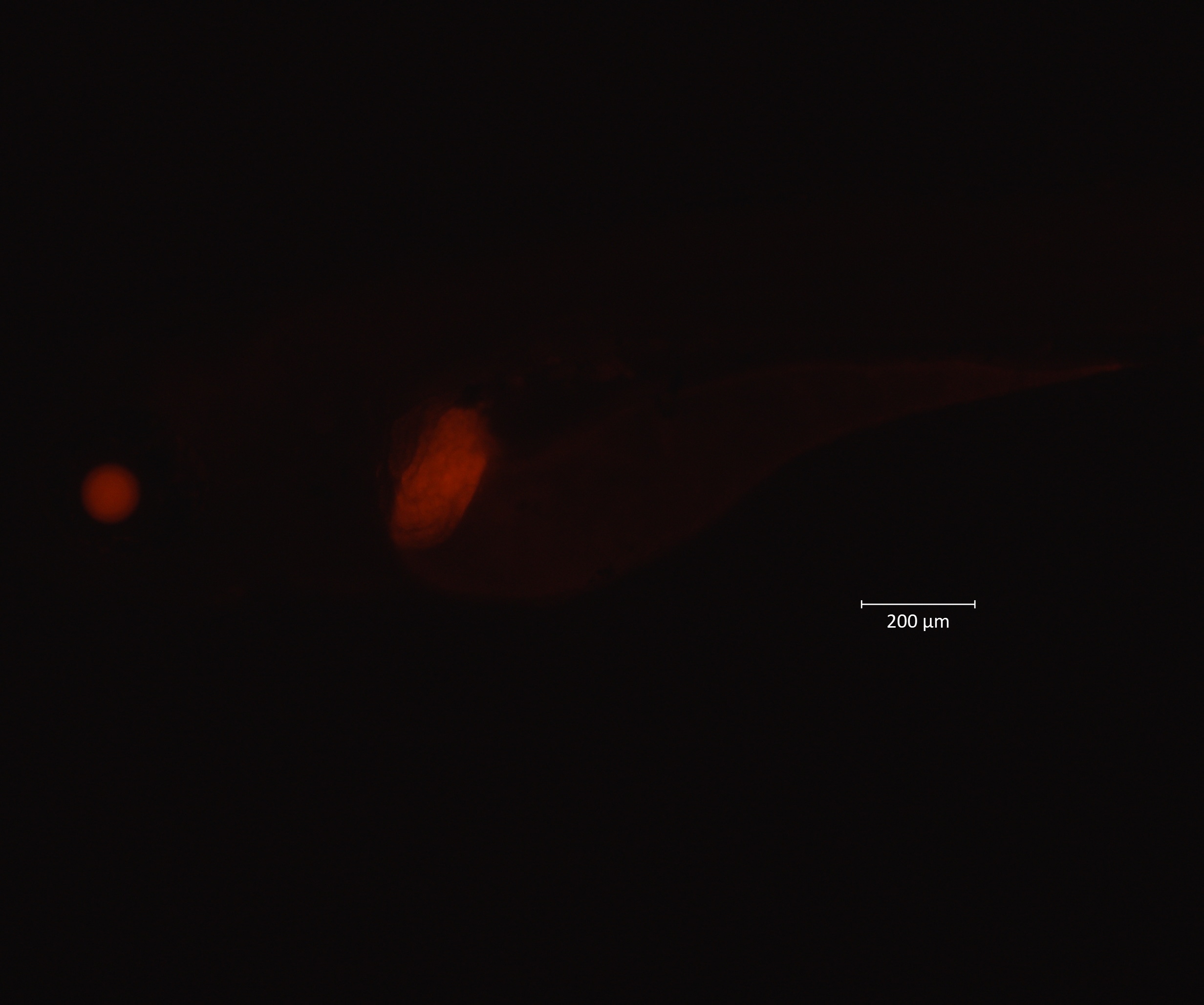

Supplement: Supplementary file 7 — Source data Fig. 5 [file 44321_2025_204_MOESM7_ESM.zip › Figure 5/5D.jpg]

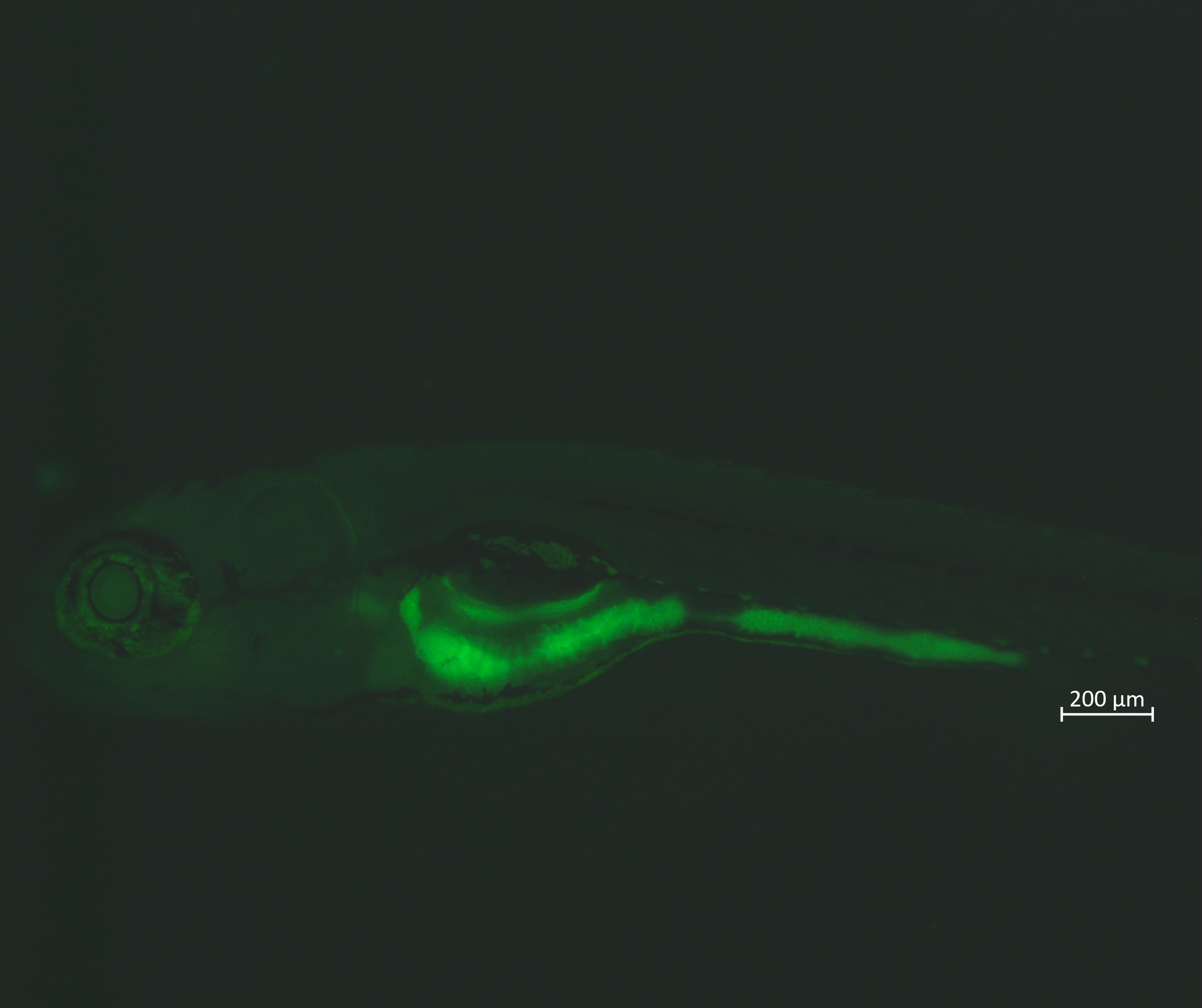

Supplement: Supplementary file 7 — Source data Fig. 5 [file 44321_2025_204_MOESM7_ESM.zip › Figure 5/5F.jpg]

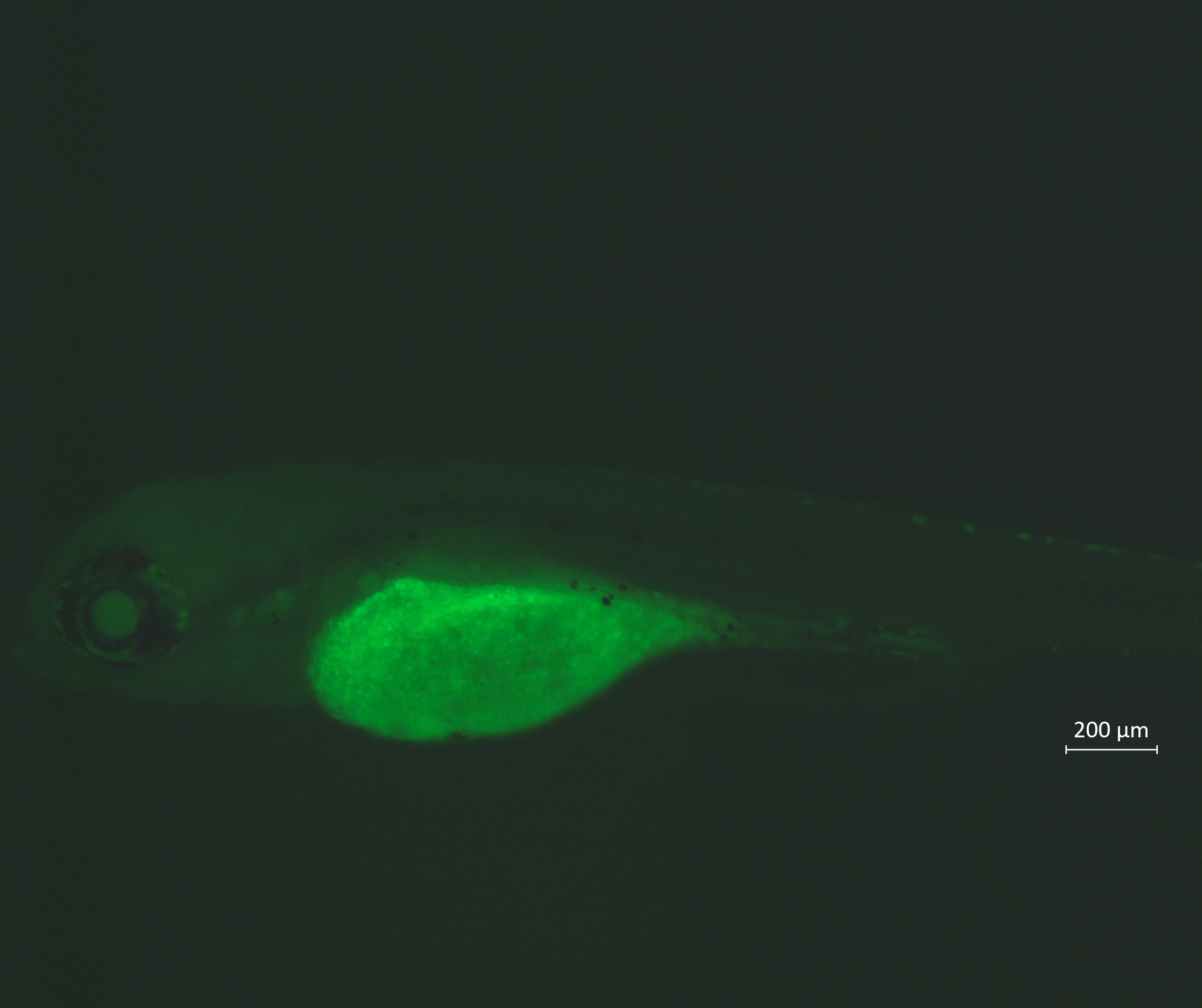

Supplement: Supplementary file 7 — Source data Fig. 5 [file 44321_2025_204_MOESM7_ESM.zip › Figure 5/5G.jpg]

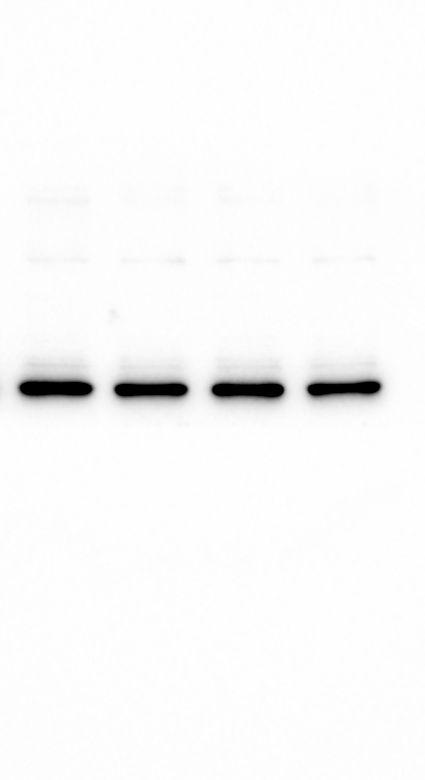

Supplement: Supplementary file 9 — Figure EV1 Source Data [file 44321_2025_204_MOESM9_ESM.zip › Figure EV1/EV1a- b actin blot.tif]

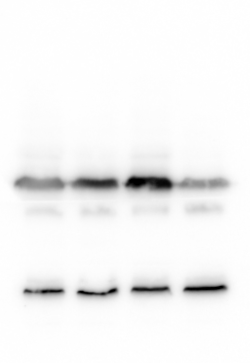

Supplement: Supplementary file 9 — Figure EV1 Source Data [file 44321_2025_204_MOESM9_ESM.zip › Figure EV1/EV1a-LC3 blot.tif]

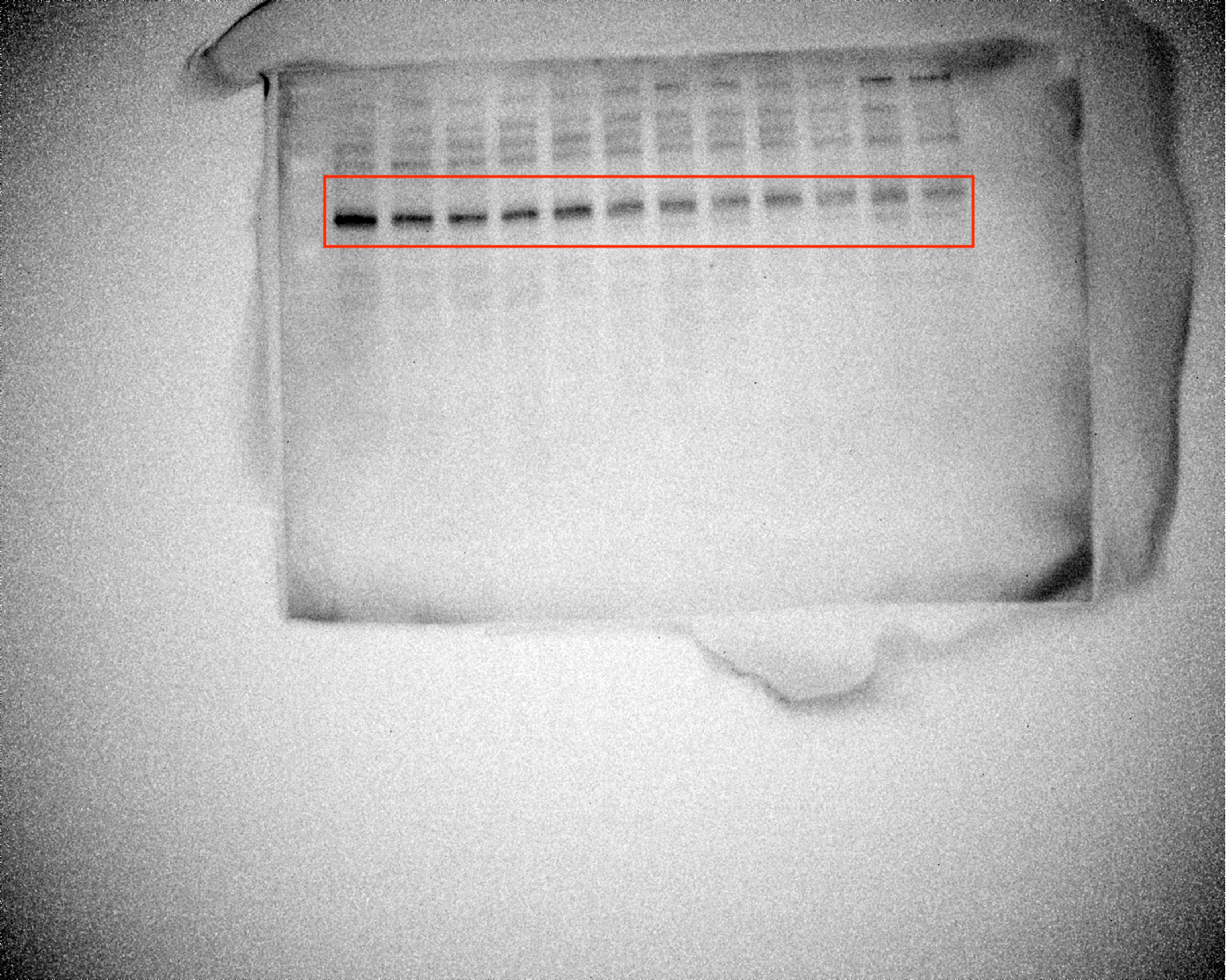

Supplement: Supplementary file 10 — Figure EV2 Source Data [file 44321_2025_204_MOESM10_ESM.zip › Figure EV2/EV2a- beta actin blot.tif]

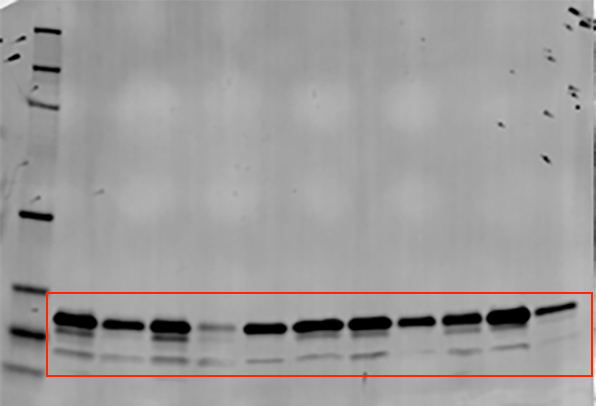

Supplement: Supplementary file 10 — Figure EV2 Source Data [file 44321_2025_204_MOESM10_ESM.zip › Figure EV2/EV2a- Lc3 blot.tif]

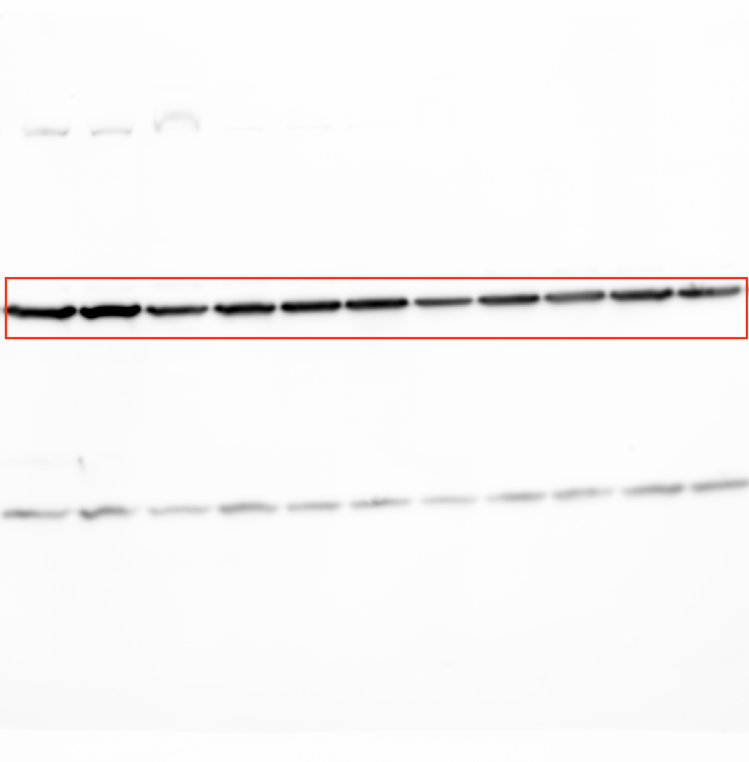

Supplement: Supplementary file 10 — Figure EV2 Source Data [file 44321_2025_204_MOESM10_ESM.zip › Figure EV2/EV2c- beta actin blot.tif]

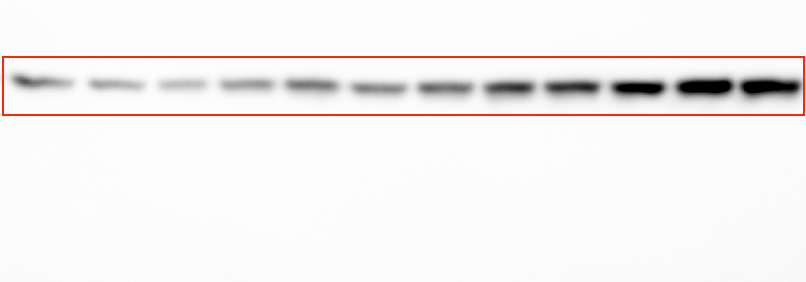

Supplement: Supplementary file 10 — Figure EV2 Source Data [file 44321_2025_204_MOESM10_ESM.zip › Figure EV2/EV2c- p62 blot.tif]

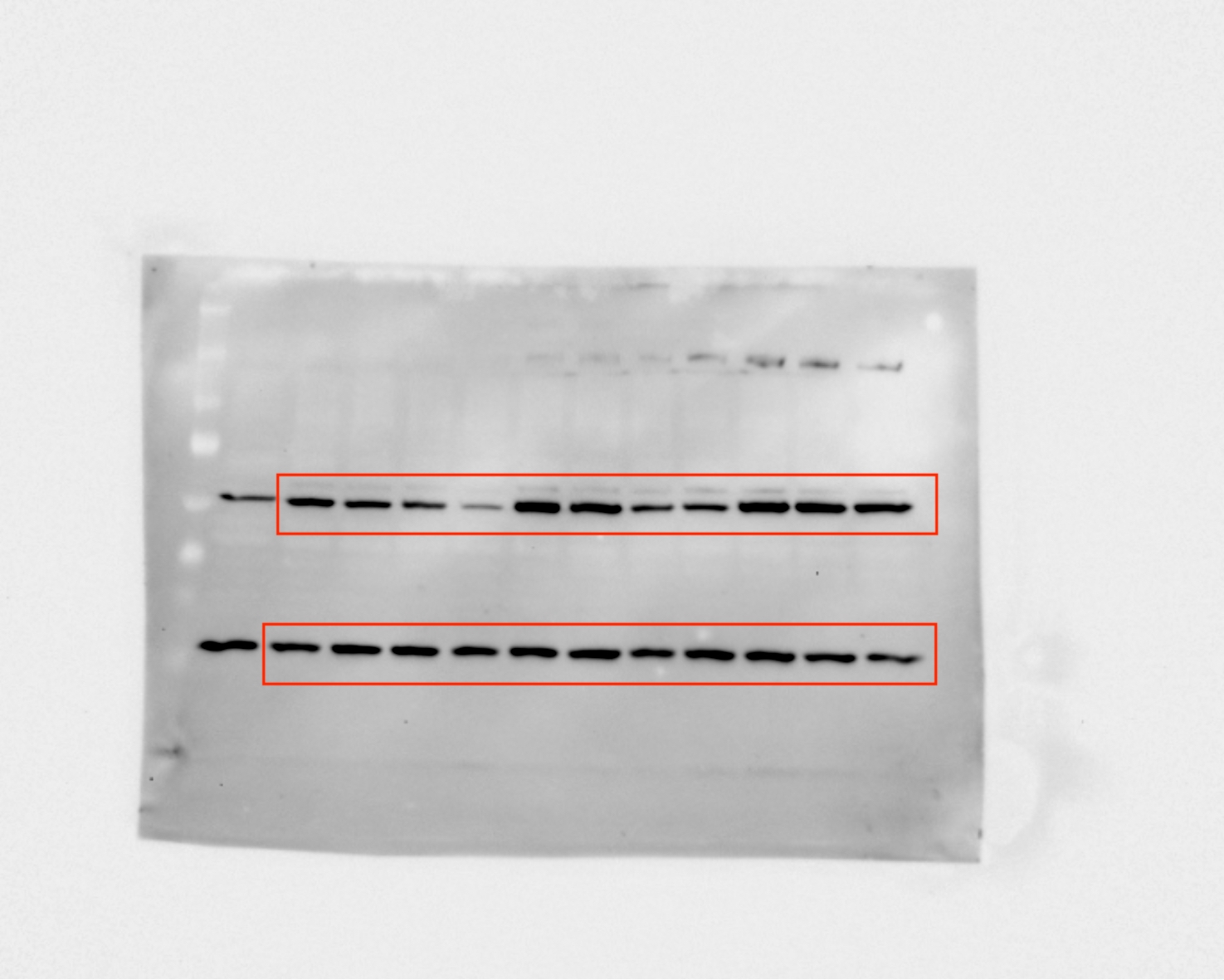

Supplement: Supplementary file 10 — Figure EV2 Source Data [file 44321_2025_204_MOESM10_ESM.zip › Figure EV2/EV2e- Atg6 and beta actin blot.jpg]
