# Supplementary material for: X-linked myopathy with excessive autophagy: characterization and therapy testing in a zebrafish model
Source: EMBO Mol Med. 2025 Feb 24;17(4):823–40. doi: 10.1038/s44321-025-00204-8 (PMC11982336; doi:10.1038/s44321-025-00204-8)
Supplement: Supplementary file 11 — Expanded View Figures [file 44321_2025_204_MOESM11_ESM.pdf]

Expanded View Figures

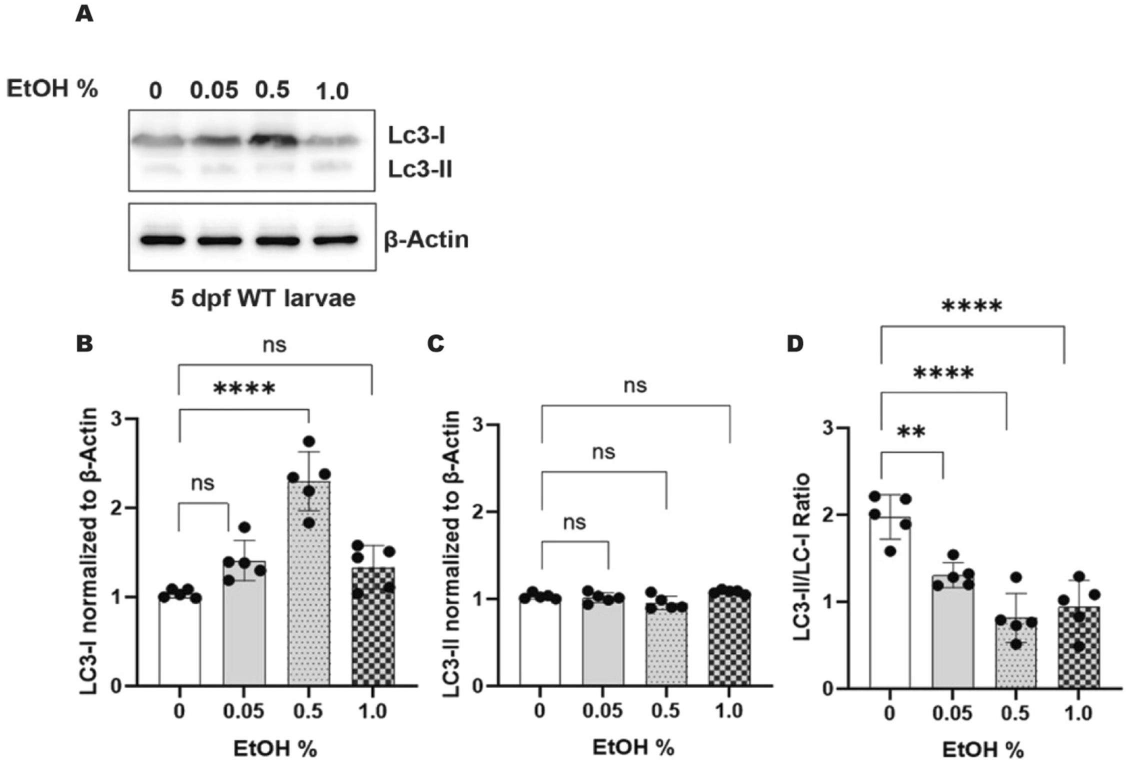

Figure EV1. Ethanol exposures increase whole-body autophagy in zebrafish.

(**A**) Western immunoblots for LC3I, LC3II, and β-actin performed on ethanol (EtOH)-treated (0, 0.05, 0.5, and 1% EtOH) zebrafish. Densitometry quantification showing the expression for (**B**) LC3I, (**C**) LC3II, and (**D**) LC3II/LC3I ratio normalized to β-actin control. One-way ANOVA are shown with *P* values indicated as the following: *$P < 0.05$; **$P < 0.01$; ***$P < 0.005$; ****$P < 0.001$. Each western blot had a total of five biological replicates. For each sample, $n = 30$ zebrafish were utilized. Data represented as mean ± SEM. Source data are available online for this figure.

 © The Author(s)

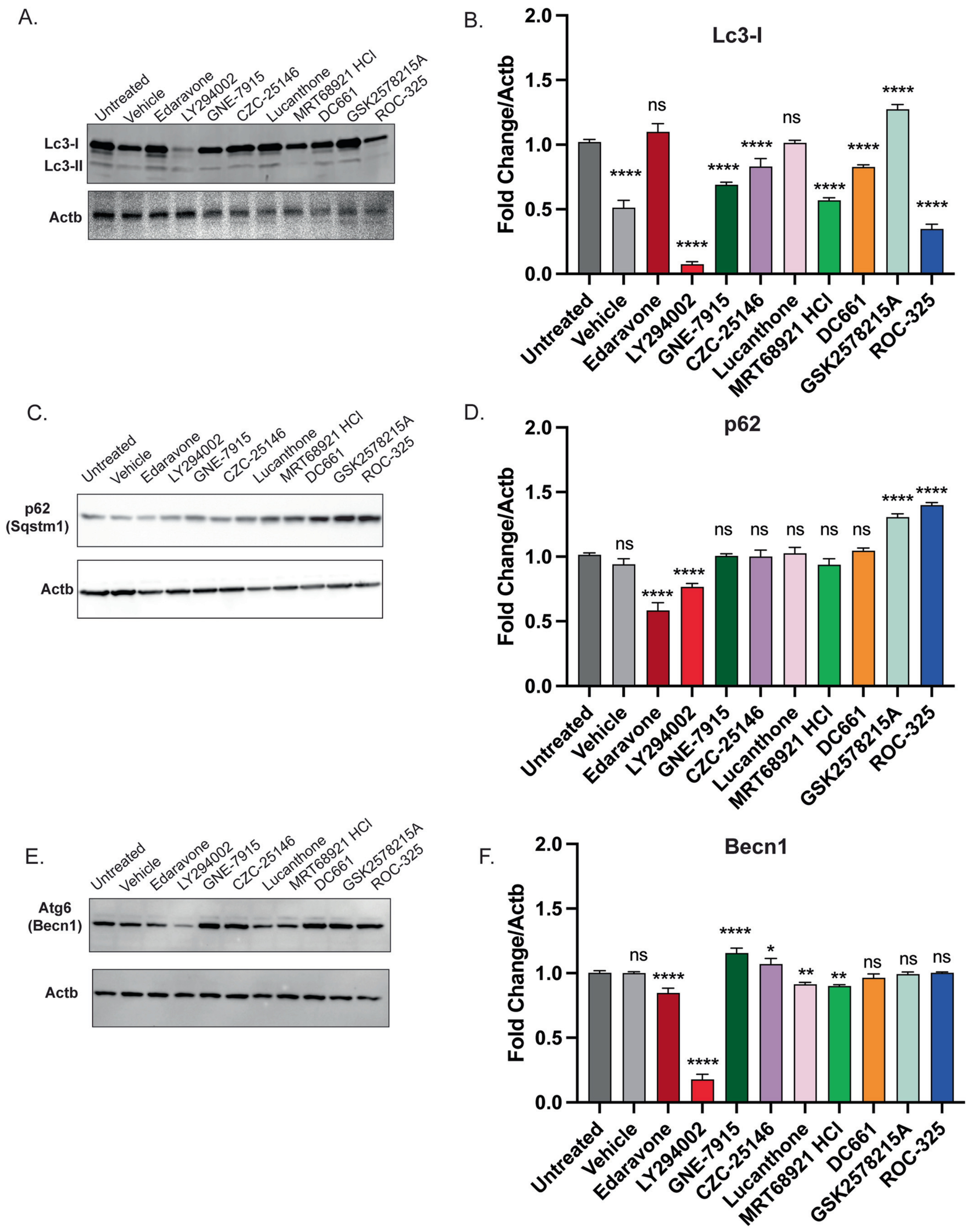
A.
Untreated
Vehicle
Edaravone
LY294002
GNE-7915
CZC-25146
Lucanthone
MRT68921 HCl
DC661
GSK2578215A
ROC-325
Lc3-I
Lc3-II
Actb
B.
Lc3-I
Fold Change/Actb
2.0
1.5
1.0
0.5
0.0
**** ns **** **** **** ns **** **** **** ****
C.
p62 (Sqstm1)
Actb
D.
p62
ns **** **** ns ns ns ns ns **** ****
E.
Atg6 (Becn1)
Actb
F.
Becn1
ns **** **** **** * ** ** ns ns ns

Figure EV2. Autophagy markers are reduced in drug-treated *vma21* mutant zebrafish.

Western immunoblots of drug-treated zebrafish as well as densitometry quantification showing the expression in drug-treated *vma21*$\Delta^{14ins21/\Delta14ins21}$ mutant homozygote mutant fish cohorts for (**A**, **B**) Lc3-I/II, (**C**, **D**) p62 (Sqstm1), and (**E**, **F**) Becn1 (Atg6) normalized to β-actin (Actb) loading controls. Nine drug compounds (Edaravone, LY294002, GNE7915, CZC-25146, Lucanthone, MRT68921 HCl, DC661, GSK2578215A, ROC-325) along with untreated and vehicle controls were evaluated. One-way ANOVA are shown with P values indicated as the following: *$P < 0.05$; **$P < 0.01$; ***$P < 0.005$; ****$P < 0.001$. Each western blot had a total of three biological replicates. For each sample, $n = 30$ zebrafish were utilized. Data represented as mean ± SEM. Source data are available online for this figure.

 © The Author(s)
